# Worldwide prevalence of rhinitis in adults: A review of definitions and temporal evolution

**DOI:** 10.1002/clt2.12130

**Published:** 2022-03-11

**Authors:** Marine Savouré, Jean Bousquet, Jouni J. K. Jaakkola, Maritta S. Jaakkola, Bénédicte Jacquemin, Rachel Nadif

**Affiliations:** ^1^ Université Paris‐Saclay UVSQ Univ. Paris‐Sud Inserm Equipe d’Epidémiologie Respiratoire Intégrative, CESP Villejuif France; ^2^ French Environment and Energy Management Agency Angers France; ^3^ Universitätsmedizin Berlin Humboldt‐Universität zu Berlin Berlin Germany; ^4^ Comprehensive Allergy Center Department of Dermatology and Allergy Berlin Institute of Health Berlin Germany; ^5^ Centre Hospitalier Universitaire Montpellier France; ^6^ MASK‐air Montpellier France; ^7^ Center for Environmental and Respiratory Health Research Faculty of Medicine University of Oulu Oulu Finland; ^8^ Medical Research Center Oulu (MRC Oulu) University of Oulu Oulu Finland; ^9^ Biocenter Oulu University of Oulu Oulu Finland; ^10^ Univ Rennes Inserm EHESP Irset (Institut de Recherche en Santé, Environnement et Travail) ‐ UMR_S 1085 Rennes France

**Keywords:** allergic rhinitis, epidemiology, non‐allergic rhinitis, prevalence, rhinitis, épidémiologie, prévalence, rhinite, rhinite allergique, rhinite non‐allergique

## Abstract

**Introduction:**

Although rhinitis is among the most common diseases worldwide, rhinitis prevalence in the general adult population is unclear and definitions differ widely.

**Objective:**

To summarize the literature on rhinitis prevalence in the general adult population and to assess: (1) the prevalence according to different rhinitis definitions overall and in different regions of the world, and (2) the evolution of rhinitis prevalence over time.

**Methods:**

We conducted an extensive literature review of publications including rhinitis prevalence using Pubmed and Scopus databases up to October 2020. We classified the definitions into three categories: unspecified rhinitis, allergic rhinitis (AR), and nonallergic rhinitis (NAR).

**Results:**

Among 5878 articles screened, 184 articles were included, presenting 156 different definitions of rhinitis. Rhinitis prevalence ranged from 1% to 63%. The overall median prevalences of unspecified rhinitis, AR and NAR were 29.4%, 18.1% and 12.0%, and they varied according to the geographical location. Rhinitis prevalence tended to increase over time.

**Conclusions:**

This review highlights the great heterogeneity of the definitions. The majority of studies had focused on AR, while only a few epidemiological data exist on NAR. We found geographical variability in rhinitis prevalence. Most of studies reported an increase of rhinitis prevalence over the last decades.

## 1 INTRODUCTION

Rhinitis is among the most common chronic diseases in the world.^1^ Rhinitis is a generic term describing nasal symptoms resulting from inflammation and/or dysfunction of the nasal mucosa.^2^ In this study, we will not consider acute infectious rhinitis that is often due to common cold or flu. The two other main phenotypes of rhinitis are allergic rhinitis (AR) and nonallergic rhinitis (NAR): AR refers to nasal symptoms that are triggered by Immunoglobulin E (IgE)‐mediated response to exposure to allergens, while NAR refers to a heterogeneous group of nasal symptoms without allergic sensitization.^3^

Several definitions of rhinitis have been used in epidemiological studies, some of them defining rhinitis as a unified entity, while others define AR and NAR separately. To date, literature reviews on rhinitis prevalence have focused solely on AR.^4^, ^5^, ^6^, ^7^ To our knowledge, the last extensive review of the literature on the prevalence of AR worldwide was conducted by the AR and its Impact on Asthma (ARIA) in 2008, and at that time the identified studies were mainly conducted in Europe or in the United States.^4^ In 2012, a literature review was conducted on the prevalence of AR outside North America and Europe^5^ and two recent literature reviews reported prevalence of AR only in China.^6^, ^7^ Therefore, there is no recent literature review on rhinitis prevalence worldwide.

Regarding the worldwide prevalence of rhinitis among children, the International Study of Asthma and Allergies in Childhood (ISAAC), which is considered as the landmark study in this field, reported in 2004 a prevalence of self‐reported current nasal symptoms of 31.7% (ranging from 11.9% to 80.6%) based on data from 97 countries.^8^ Among adult populations, the largest international multicenter study is the European Community Respiratory Heath Survey (ECRHS), and it estimated a median prevalence of self‐reported nasal allergies of 20.9% (ranging from 9.5% to 40.9%) in 1995.^9^ That study did not estimate the overall prevalence of rhinitis nor that of NAR, and even though it included altogether 20 countries, they were mainly from the same geographical area, that is from the Western Europe. It is commonly proposed in the literature that rhinitis prevalence has increased in recent decades,^4^ but there is no literature review on this topic.

In summary, many different definitions of rhinitis have been applied, and the majority of the studies have focused on AR, with only a few studies focusing on NAR. Moreover, the estimates of rhinitis prevalence in adults in different parts of the world are still poorly known.

The objective of the present study was to conduct an extensive literature review on rhinitis prevalence in general adult population around the world. This review describes rhinitis prevalence according to different definitions and worldwide. It also aims to describe the temporal evolution of rhinitis prevalence.

## 2 METHODS

**FIGURE 1:**
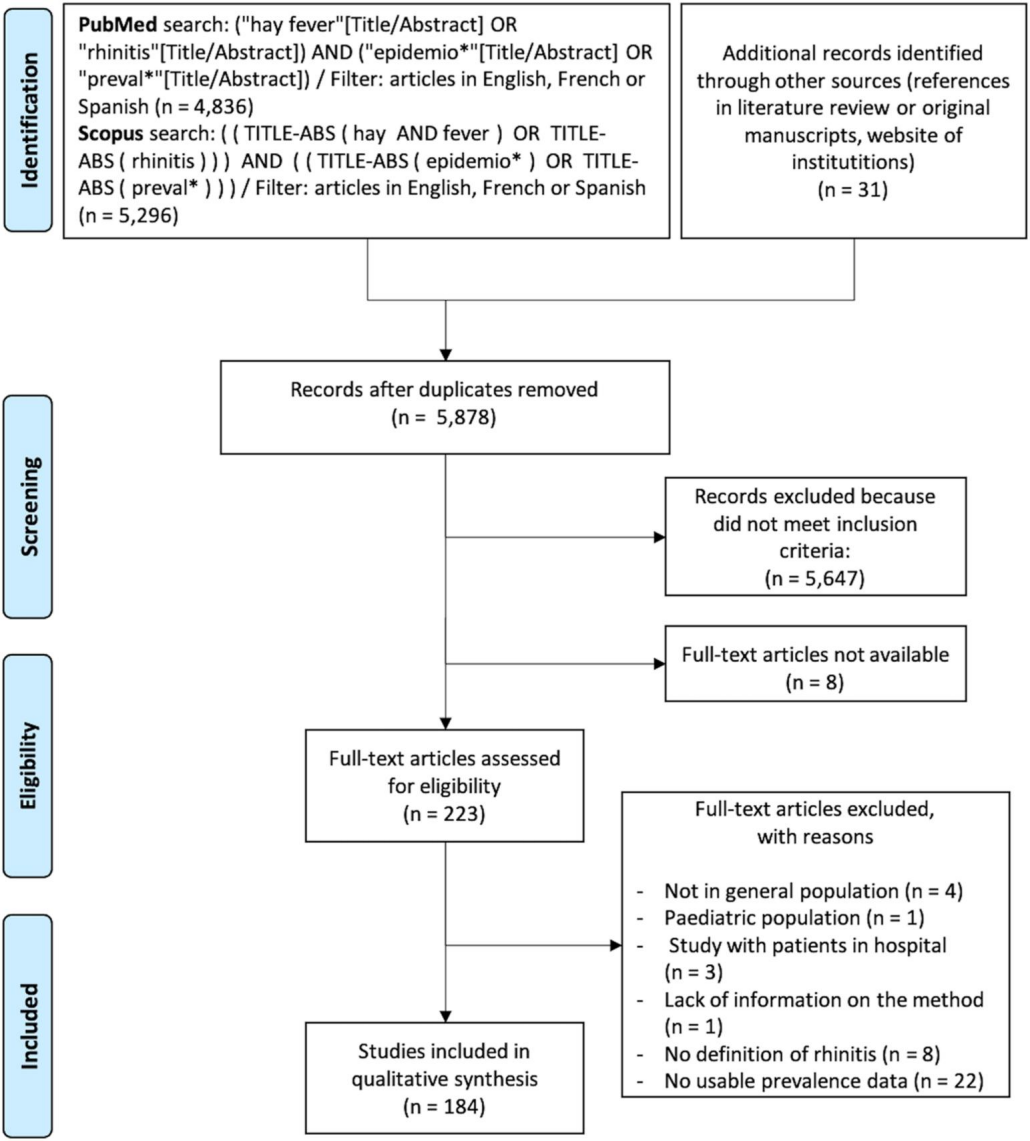
Flow diagram showing selection of articles reviewed

The present literature review on rhinitis prevalence was performed according to the four‐phase flow diagram presented in the PRISMA statement as follows: identification, screening, defining eligibility, and inclusion (Figure 1).

### 2.1 Information sources, search strategy, selection process and eligibility criteria

The literature search used the computerized bibliographic databases PubMed and Scopus and included all the existing literature up to October 13, 2020.

We searched for the terms “rhinitis” or “hay fever” combined with “epidemio*" or “preval*” in the title or abstract. The only limitation we applied was that the studies had to be written in English, French, or Spanish language. Additional records from the references of the literature reviews and of the eligible publications identified in the search were included. The articles were first screened based on their title and abstract, and then articles of interest were read entirely to assess their eligibility.

Articles were selected according to the following criteria: the articles were required to (1) be an original epidemiological study, (2) include prevalence data on rhinitis, AR and/or NAR, (3) be based on a general adult population or a population including all ages. As this study focuses on rhinitis prevalence in general population, we excluded studies focusing only on specific subpopulations such as children, students, farmers, exclusively in men, or women. We also excluded studies that were conducted in specific health centers or allergy clinics and studies that did not contain any information on the definition of rhinitis, or that did not provide any calculable rhinitis prevalence in the general population.

### 2.2 Data items

The following data were collected: (1) the year of publication, (2) the country where the study was conducted, (3) the period during which the study was conducted, (4) the size and age range of the studied population, (5) the study design, including cross‐sectional and/or longitudinal, (6) the method applied to assess the prevalence, including interview or self‐report, and (7) the definition applied for rhinitis.

When not directly reported in the article, the prevalence was calculated based on the available data, applying the following formula: the number of participants with rhinitis divided by the total number of participants. We reported the prevalence in adults only if available, otherwise we reported the prevalence of the population including adults and children.

#### 2.2.1 Classification of the data

We have categorized the different definitions of rhinitis into three categories as follows: “unspecified rhinitis” refers to a definition of rhinitis that did not specify whether it was allergic or nonallergic; AR refers to a definition that included the term “AR” and/or “hay fever” and/or included exposures known to induce an allergic inflammation of the airways, such as pollen, furry animals, or house dust mites; and/or assessment of allergic sensitization by skin tests and/or serum specific IgE; NAR refers to a definition that excluded AR and/or referred to triggers known to induce NAR, such as cold and dry air, temperature change, airborne chemical irritants, spicy food, alcoholic beverages, exercise, use of tobacco, anti‐inflammatory drugs, stress, and/or printer ink. In addition, we have further subcategorized unspecified rhinitis, AR and NAR according to the method on which the definition was based: “symptoms‐based definition” which refers to a definition that is based on a question asking from the participant himself/herself about the presence of rhinitis or nasal symptoms; while “doctor diagnosis‐based definition” of rhinitis was based on asking a question referring to a previous self‐reported medical diagnosis of rhinitis or on a diagnosis made by a physician for the study. Finally, “IgE/SPT‐based definition” refers to a definition based on either of the two previously mentioned definitions in combination with the assessment of the IgE‐mediated sensitization with measurements of specific IgE and/or skin prick tests (SPTs). Participants with positive SPTs and/or positive specific IgE were defined as AR IgE/SPT‐based definition, those with negative IgE/SPT results were defined as NAR IgE/SPT‐based definition. Finally, we subcategorized unspecified rhinitis, AR and NAR into (1) “ever rhinitis” that refers to the presence of rhinitis ever in the study participant's lifetime (i.e., lifetime prevalence), and (2) “current rhinitis” that refers to the presence of rhinitis at the time of the study or in the last few months (i.e., point or months/1‐year period prevalence). The details of the definitions and their categorization are presented in Appendix 2 in Supporting Information S2.

### 2.3 Statistical methods

Mean prevalences were calculated for the unspecified rhinitis, AR, and NAR applying Microsoft Excel®. For each definition, we calculated the crude mean, median and confidence intervals based on binomial distribution with R 4.3. To compare rhinitis prevalence according to different regions of the world, prevalences were first grouped by country and then the countries were stratified by continent.

To study the evolution of rhinitis prevalence over time, we selected studies that had repeated measures of the prevalence using the same definition and comparable populations over time.

As a complementary analysis, we calculated the rhinitis prevalences based solely on the ISAAC and ECRHS questions that are from the two largest international studies on rhinitis and that were used in several studies included in the present literature review.

We also conducted sensitivity analyses with more restrictive definitions and populations to obtain less biased prevalences of unspecified rhinitis, AR and NAR in the general adult populations. We conducted sensitivity analyses based on the following two criteria:

(1) To include only more “reliable” definitions of rhinitis, we excluded those studies in which the definition was/had been based on: (a) questions that did not exclude explicitly common cold or flu; (b) questions that included other allergic diseases in the same item, such as conjunctivitis or eczema; (c) questions that were limited to the time of the survey; (d) questions that did not specify when rhinitis was or had been present, and (e) questions that focused solely on specific sub‐phenotypes of rhinitis such as rhino‐conjunctivitis, seasonal AR, or peri‐annual rhinitis.
(2) To have a more homogeneous adult population, we excluded studies that reported prevalences based on populations: (a) that combined adults and children (i.e., under the age of 16 years), and (b) that included the elderly only (i.e., above the age of 60 years).

## 3 RESULTS

After excluding duplicates, a total of 5878 records were identified. Altogether 184 articles were included in the present review (Figure 1, Appendix 1 in Supporting Information S1) with 426 reported prevalences of unspecified rhinitis, AR or NAR.

### 3.1 Rhinitis definitions and estimates for prevalence

A total of 156 different definitions of rhinitis were identified: 58 for unspecified rhinitis, 86 for AR, and 12 for NAR (see Appendix 2 in Supporting Information S2). Table 1 describes the rhinitis prevalences for these three main categories and their subcategories. The median rhinitis prevalences were: 29.4% (ranging from 1.1% to 63.3% based on 103 reported prevalences) for unspecified rhinitis, 18.1% (ranging from 1.0% to 54.5% based on 310 reported prevalences) for AR, and 12.0% (ranging from 4.0% to 31.4% based on 13 reported prevalences) for NAR. The median prevalence of current AR was 21.6% based on symptoms‐based definition and 16.4% based on IgE/SPT‐based definition. For NAR, the median prevalence was 16.4% based on symptoms‐based definition and 31.4% based on IgE/SPT‐based definition. In adults of all ages, NAR was reported in 24.4%–67.1% of participants with rhinitis.^10^, ^11^, ^12^, ^13^, ^14^, ^15^, ^16^, ^17^, ^18^, ^19^, ^20^

**TABLE 1:** Rhinitis prevalences according to the different definitions

	*n*	Mean (%)	Med (%)	SD (%)	CI_95%_	Min. (%)	Max. (%)
All definitions							
Unspecified rhinitis	103	27.0	29.4	14.0	(24.3, 29.7)	1.1	63.3
AR	311	19.1	18.1	10.2	(18.0, 20.3)	1.0	54.5
NAR	13	14.6	12.0	8.3	(9.6, 19.6)	4.0	31.4
Symptoms‐based definitions							
Unspecified rhinitis							
Ever	20	28.1	30.4	12.8	(22.1, 34.0)	10.8	50.2
Current	68	30.8	32.3	12.5	(27.8, 33.8)	4.1	63.3
AR							
Ever	42	21.4	20.7	10.9	(18.0, 24.8)	4.2	52.0
Current	145	23.5	21.6	9.7	(21.9, 25.1)	3.6	54.5
NAR							
Ever	1	7.9					
Current	6	10.0	16.4	4.4	(5.4, 14.6)	4.0	16.4
Doctor diagnosis‐based definitions							
Unspecified rhinitis							
Ever	14	9.0	9.1	3.3	(7.2, 10.9)	2.5	14.0
Current	1	1.1					
AR							
Ever	53	12.8	13.0	6.9	(10.9, 14.7)	1.0	30.9
Current	28	7.9	7.9	1.4	(7.4, 8.5)	3.9	11.4
IgE/SPT‐based definitions							
AR							
Ever	3	15.5	28.5	11.3		8.9	28.5
Current	39	17.4	16.4	7.3	(15.1, 19.8)	3.7	44.2
NAR							
Ever	0						
Current	6	20.4	31.4	8.5	(11.5, 29.3)	5.5	31.4

Abbreviations: AR, allergic rhinitis; CI, confidence interval; Current, combination of point and period prevalences; Ever, Lifetime prevalences; Max, highest reported prevalence; Med, Median; Min, lowest reported prevalence; *n*: number of reported prevalences; NAR, non‐allergic rhinitis; SD, standard deviation.

Using the ECRHS definition, the median current AR prevalence was 22.7% (ranging from 7.0% to 47.5% based on 87 reported prevalences). Using the ISAAC definitions, the median prevalence was 32.7% (ranging from 11.4% to 38.0% based on 6 reported prevalences) for ever rhinitis, and 30.8% (ranging from 10.4% to 38.3% based on 15 reported prevalences) for current rhinitis.

Sensitivity analyses were based on 63 “reliable” definitions of rhinitis, while 333 prevalences were excluded: 284 due to the definition criteria, 49 due to the age range studied. A total of 93 reported prevalences were included, the median prevalences being 32.4% (ranging from 10.4% to 54.1% based on 27 reported prevalences) for unspecified rhinitis, 17.1% (ranging from 1.0% to 44.2% based on 63 reported prevalences) for AR, and 16.4% (ranging from 6.5% to 23.5% based on 3 reported prevalences) for NAR (Table S1 in Supporting Information S3).

### 3.2 Rhinitis prevalences worldwide

**FIGURE 2:**
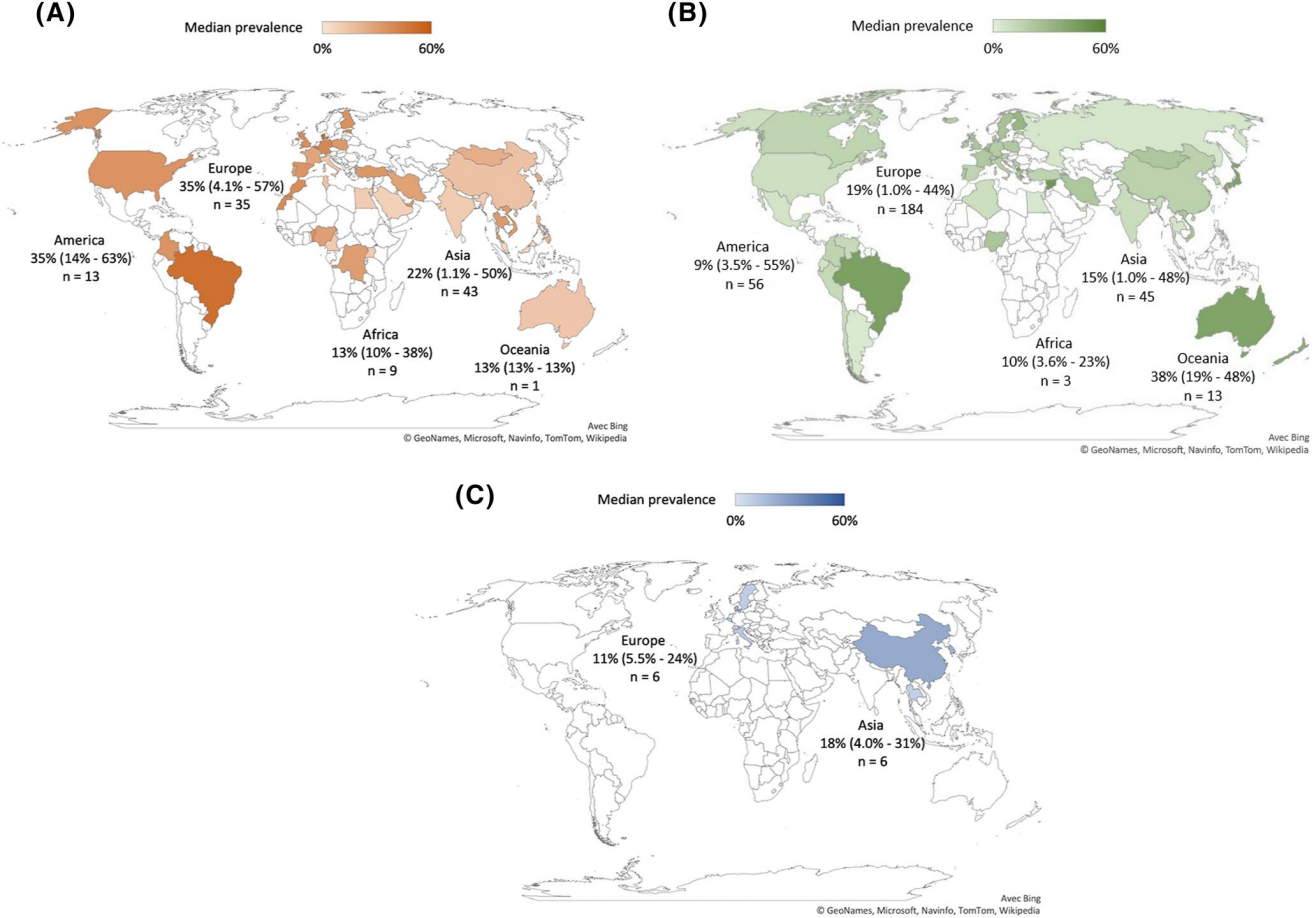
Rhinitis prevalences in different regions of the world. (A) Unspecified rhinitis; (B) allergic rhinitis; (C) non‐allergic rhinitis. The data for continents are presented as follows: median (minimum reported–maximum reported), *n* = number of reported prevalences. Colored countries are those for which data are available. The darkest countries are those for which the median prevalence is the highest

World maps showing the average prevalences for the unspecified rhinitis, AR and NAR are presented in Figure 2. There was a wide variation in the reported prevalences even within the same continent for all categories of rhinitis. Details of the reported prevalences by country are presented in Table S2 in Supporting Information S3. Prevalence of unspecified rhinitis ranged from 10.4% to 37.8% for Africa, from 14.0% to 63.3% for America, from 1.1% to 50.2% for Asia, from 4.1% to 56.6% for Europe, and 13.2% for Oceania (based on a single study^21^). Prevalence of AR ranged from 3.6% to 22.8% for Africa, from 3.5% to 54.5% for America, from 1.0% to 47.9% for Asia, from 1.0% to 43.9% for Europe, and from 19.2% to 47.5% for Oceania. Only a few prevalences of NAR have been reported, and no data was found from America, Africa or Oceania; for Asia, six reported prevalences were found ranging from 4.0% to 31.4%, and for Europe, six prevalences were found ranging from 5.5% to 23.5%.

### 3.3 Evolution in the rhinitis prevalence over time

**FIGURE 3:**
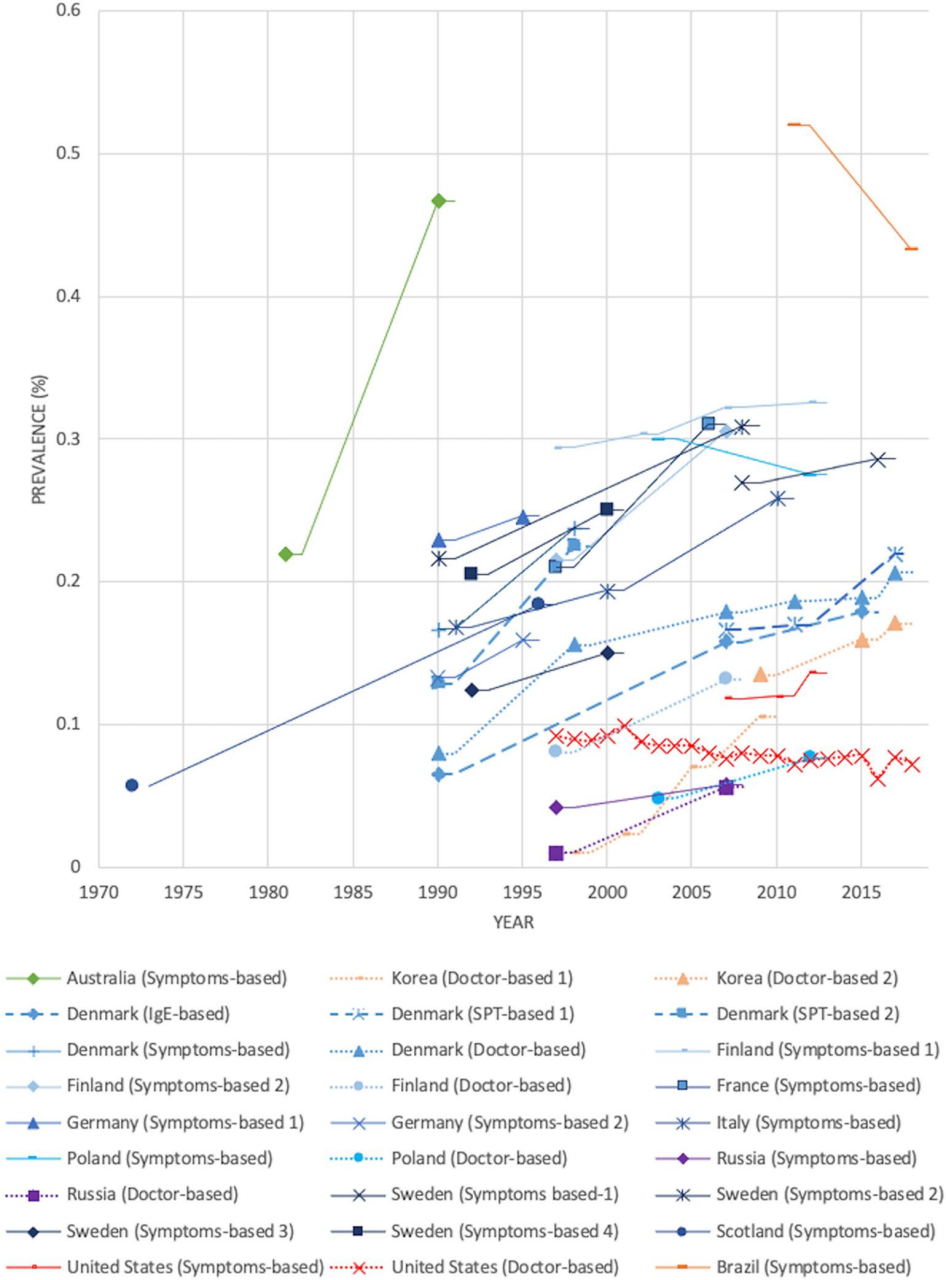
Evolution of allergic rhinitis prevalence worldwide over time

The evolution over time of rhinitis prevalence within the same population or similar populations and applying the same definition in the same geographical region are presented in Figure 3. Table S3 in Supporting Information S3 provides details of the prevalences by country.

In the America, in Brazil a 10% decrease in the rhinitis prevalence was reported between 2011 and 2018.^22^ In the United States, the National Health and Nutrition Examination Survey (NHANES) reported that episodes of hay fever in the past 12 months increased from 11.8% to 13.6% between 2007 and 2012. In the National Health Interview Survey (NHIS), the prevalence of AR decreased from 9.3% in 1997 to 7.3% in 2018.

In Asia, the Korean National Health and Nutrition Examination Survey reported that the prevalence of AR increased between 1998 and 2017 from 1.0% to 17.1%.^23^, ^24^

In Europe, studies from Denmark,^25^, ^26^ Finland,^27^, ^28^ France,^29^, ^30^ Germany,^31^ Italy,^32^ Russia,^27^ Scotland,^33^ and Sweden^34^, ^35^, ^36^ reported an increase in the prevalence of rhinitis. In Poland, the results varied depending on the definition used: between 2003 and 2012 a slight decrease was observed for rhinitis based on “Problem with sneezing or a runny or blocked nose or itchy eyes in April, May, June, or July” and “In last 12 months, have you had a problem with sneezing or a runny or blocked nose or itchy eyes when you (your child) did not have a cold or flu?”.^37^ In the same study, an increase from 4.8% to 7.7% was observed in rhinitis defined by “Has a physician ever told you that you have hay fever?”.^37^

For Oceania, one study in Australia showed that between 1981 and 1990 the hay fever prevalence increased from 21.9% to 46.7%.^38^ No studies were found on the evolution of rhinitis prevalence over time from the African continent.

## 4 DISCUSSION

In this study, we identified a total of 184 articles that included data on the rhinitis prevalence among adults, contributing altogether 156 different definitions of rhinitis. Depending on the definition used and the geographical area studied, rhinitis prevalence ranged from 1% to over 60%. The median worldwide prevalences of unspecified rhinitis, AR and NAR were 29.4%, 18.1%, and 12.0%. A geographical variability was observed in the prevalences of subtypes of rhinitis. Irrespective of the definition used, most of the studies reported an increase in the rhinitis prevalence over the last decades.

We conducted an extensive literature search using the most commonly used keywords for rhinitis and AR, namely “rhinitis” and “hay fever”. In an additional search, we also considered to use keywords “nose symptoms” or “nasal symptoms” as synonyms for rhinitis, but the results of these searches only provided additional articles that referred to differential diagnoses of rhinitis, such as COVID‐19 or nasal polyps. Therefore, these terms were not included in the main search. As we have limited this review to articles published in English, French and Spanish, it is possible that we may have omitted some prevalence data from articles published in other languages.

We found a total of 156 rhinitis definitions in the present literature review, so we decided to classify them into three distinct categories: unspecified rhinitis, AR and NAR. To our knowledge, this is the first time that such categorization of rhinitis has been applied within an extensive literature review, usually all definitions of rhinitis being considered together within one term. We focused on a classification of rhinitis as an entity or according to its two main phenotypes: AR or NAR. Prevalences of other sub‐phenotypes of rhinitis, such as rhino‐conjunctivitis, are also of interest per se and should be assessed in more specific reviews.

Overall, we found a median prevalence of 29% for unspecified rhinitis, which is close to the prevalence of 31.7% reported for children aged 13–14 years in the ISAAC phase 3 study.^8^ However, a large variability was observed between the studies of rhinitis prevalence. This variability is probably partly explained by heterogeneity in the definitions used in the studies. Indeed, within the same population the prevalence was found to vary greatly depending on the definition used: for example, in the study by Bauchau et al. rhinitis prevalence in Belgium varied by more than 30% depending on whether rhinitis was defined based on doctor diagnosis or on reported symptoms.^39^ Considering all the studies, we found that the average prevalence of doctor diagnosis‐based definitions was lower compared to the other definitions. This could be partly explained by the fact that rhinitis prevalence is often underestimated when based on the doctor diagnosis. Indeed, it is recognized that patients with rhinitis often tend to self‐diagnose their rhinitis and treat themselves,^40^ and those who consult a doctor are likely to have more often moderate to severe rhinitis.^41^ In addition, access to health professionals may be heterogeneous according to the geographic location, healthcare system, or socio‐economic status of the patient. Large variabilities in reported prevalences were also observed for AR and NAR. The median prevalence of AR obtained in this literature review based on all definitions was 18%, with a higher average prevalence being detected with the symptoms‐based definitions compared to the IgE/SPT‐based definitions. We obtained a median prevalence of NAR of 12%, which is lower than the prevalence obtained for AR. Nevertheless, based on our literature review, compared to AR, NAR could represent 20%–80% of rhinitis cases in adults. As only 13 studies had evaluated the prevalence of NAR, it is rather difficult to make conclusions on the “true” prevalence of NAR.

The wide variability in definitions of rhinitis detected in this extensive review makes it recommendable to try to achieve a consensus on which definitions should be used in epidemiological studies. However, to date, no such consensus has been reached. In our opinion, it would be best to define unspecified rhinitis in epidemiological studies applying a question that is easily understandable by all participants, that refers to the main symptoms of rhinitis, and that does not include specific medical terms. The AR and Its Impact on Asthma (ARIA) recommended to use a question including symptoms of “sneezing, runny nose and/or blocked nose when the patient does not have a cold”.^3^ The definitions of current and ever rhinitis should not include any ambiguous terms, such as “often,” “several,” or “most of the time.” Instead, it would be better to refer to clear time periods, for example, “in the last 12 months” or “during your lifetime.” The two questions in the ISAAC study^42^ comply with these recommendations, and, in our opinion, are accurate questions to define current and ever unspecified rhinitis. In clinical practice, to define AR and NAR, the measurement of specific IgE or SPTs combined with a medical history by the doctor is “the gold standard.” However, as these diagnostic tools are not always easily available in large epidemiological studies, it could be useful to have a proxy for AR and NAR defined based on questionnaire. We have recently shown that a combination of the ISAAC and ECRHS definitions could be a suitable proxy.^43^ Another way to define AR and NAR in the absence of IgE/SPT measurements would be to use questions referring to the main triggers of nasal symptoms. Specific allergens triggering rhinitis symptoms, such as pollen or animal exposure, or the presence of eye symptoms in combination with rhinitis symptoms have been shown to have a moderately high positive predictive value for AR in participants with SPT positivity to common aeroallergens.^44^ These triggers as well as associated eye symptoms emerged as important discriminative variables in a clustering analysis that was performed without any a priori hypothesis to identify AR and NAR.^45^ However, an expert consensus and/or methodological research comparing different definitions with a clinical diagnosis of rhinitis is needed to establish the best definitions for rhinitis, AR and NAR, to be used in epidemiological studies.

In this study, we carried out a sensitivity analysis including only definitions that were judged as “reliable” based on our proposed criteria for unspecified rhinitis, AR and NAR. Nevertheless, even in this sensitivity analysis a great heterogeneity was observed between the different prevalences. Moreover, using identical definitions, for example, ISAAC or ECRHS definitions, rhinitis prevalence still varies between different countries of the world.

This geographical variability in rhinitis prevalence could be partly explained by different environmental exposures, such as different species of pollen, different lifestyle factors, including dietary habits and keeping of pets indoors, or different host risk factors.^5^ As in this study, we have reported rhinitis prevalences without considering these risk factors, these hypotheses need to be tested in future studies. It should be noted that these factors were rarely considered and/or investigated in the studies that were identified by this literature review.

Rhinitis appears to be a common disease in the European and American countries, which is consistent with some previous reports.^4^, ^5^ In other parts of the world, far fewer studies have been reported on the prevalence of different types of rhinitis, which makes it more difficult to calculate reliable estimates and make firm conclusions. It is noteworthy that the global distribution of rhinitis prevalence was found to be similar to that of global distribution of asthma prevalence, for which there is more studies.^46^ Further studies that apply standardized definitions of rhinitis in different parts of the world would throw more light on potential “true” differences in rhinitis prevalence and on potential causes underlying such differences.

Our extensive review showed that most studies reported an increase in rhinitis prevalence over time. One explanation could be that the awareness of rhinitis has improved in parallel in different populations around the world due to advertising of over‐the‐counter medicines for rhinitis, and with the development of the Internet making it easier to get information on rhinitis. In addition, medical examinations may have become better accessible and thus, the rhinitis diagnosis may also be more easily accessible. In any case, rhinitis prevalence seems to have increased in many parts of the world, whatever the definition of rhinitis. Concerning the trend of increasing prevalence of different types of rhinitis, potential impact of risk factors, including global warming, changes in seasonal patterns, and air pollution, have been proposed.^47^ However, only few studies have investigated potential impact of such factors on rhinitis prevalence over time.

In conclusion, our review highlights the lack of consensus on which questions the phenotypes of rhinitis should be based. This review also found a geographical variability of rhinitis prevalences, and pointed out the scarcity or even inexistence of data from some parts of the world. While these points could appear as not being novel, there is no recent literature review on this topic that would summarize these facts. The present extensive review underlines the urgent need for a consensus on standardized definitions for rhinitis to be used in epidemiological studies. Finally, for the first time in a literature review, this study shows that rhinitis prevalence in adults is increasing.

## CONFLICT OF INTERESTS

The authors declare that they have no known competing financial interests or personal relationships that could have appeared to influence the work reported in this study.

## AUTHOR CONTRIBUTIONS

**Marine Savouré:** Conceptualization; Lead, Data curation; Lead, Formal analysis; Lead, Funding acquisition; Lead, Investigation; Lead, Methodology; Lead, Visualization; Lead, Writing – original draft; Lead, Writing – review & editing; Lead, **Jean Bousquet:** Conceptualization; Supporting, Methodology; Supporting, Visualization; Supporting, Writing – original draft; Supporting, Writing – review & editing; Equal, **Jouni J.K. Jaakkola:** Conceptualization; Supporting, Methodology; Supporting, Visualization; Supporting, Writing – original draft; Supporting, Writing – review & editing; Equal, **Maritta Jaakkola:** Conceptualization; Supporting, Methodology; Supporting, Visualization; Supporting, Writing – original draft; Supporting, Writing – review & editing; Equal, **Benedicte Jacquemin:** Conceptualization; Equal, Methodology; Equal, Supervision; Equal, Validation; Equal, Visualization; Equal, Writing – original draft; Equal, Writing – review & editing; Equal, **Rachel Nadif:** Conceptualization; Equal, Methodology; Equal, Resources; Lead, Supervision; Equal, Validation; Equal, Visualization; Equal, Writing – original draft; Equal, Writing – review & editing; Equal.

## Supporting information

Supporting Information S1

Supporting Information S2

Supporting Information S3
