# Supplementary material for: Worldwide prevalence of rhinitis in adults: A review of definitions and temporal evolution
Source: Clin Transl Allergy. 2022 Mar 11;12(3):e12130. doi: 10.1002/clt2.12130 (PMC8967272; doi:10.1002/clt2.12130)
Supplement: Supplementary file 1 — Supporting Information S1 [file CLT2-12-e12130-s003.docx]

# Online supplementary material

**Worldwide prevalence of rhinitis in adults: a review of definitions and temporal evolution**

# Marine Savouré *et al.*

**Appendix**

Appendix 1: Details of the selected studies (excel file)

Appendix 2: Details of the definitions (excel file)

**References included in the literature review**

1. Abdulrahman H, Hadi U, Tarraf H, Gharagozlou M, Kamel M, Soliman A, Hamad WA, Hanna KM, Mostafa BE, Omrani M, Abdelmotal A, Moukarzel N. Nasal allergies in the Middle Eastern population: results from the “Allergies in Middle East Survey.” Am J Rhinol Allergy 2012; 26 Suppl 1: S3-23.

2. Abong JM, Kwong SL, Alava HDA, Castor MAR, De Leon JC. Prevalence of allergic rhinitis in Filipino adults based on the National Nutrition and Health Survey 2008. Asia Pac Allergy 2012; 2: 129–135.

3. Abramson M, Kutin J, Czarny D, Walters EH. The prevalence of asthma and respiratory symptoms among young adults: is it increasing in Australia? J Asthma 1996; 33: 189–196.

4. Abramson MJ, Schindler C, Schikowski T, Bircher AJ, Burdet L, Gerbase MW, Imboden M, Rochat T, Schmid-Grendelmeier P, Turk AJ, Zemp E, Künzli N, Probst-Hensch N. Rhinitis in Swiss adults is associated with asthma and early life factors, but not second hand tobacco smoke or obesity. Allergology International 2016; 65: 192–198.

5. Abramson M, Kutin JJ, Bailey M, Raven J, Dunster K, Rolland J, Czarny D, Walters EH. Nasal allergies hayfever among young adults in Melbourne, Australia. Allergology International 1997; 46: 213–219.

6. Acharya D, Bajgain BB, Yoo S-J. Factors Associated with Atopic Dermatitis and Allergic Rhinitis among Residents of Two Municipal Areas in South Korea. Medicina (Kaunas) 2019; 55.

7. Ahn J-C, Kim J-W, Lee CH, Rhee C-S. Prevalence and Risk Factors of Chronic Rhinosinusitus, Allergic Rhinitis, and Nasal Septal Deviation: Results of the Korean National Health and Nutrition Survey 2008-2012. JAMA Otolaryngol Head Neck Surg 2016; 142: 162.

8. Al-Digheari A, Mahboub B, Tarraf H, Yucel T, Annesi-Maesano I, Doble A, Lahlou A, Tariq L, Aziz F, El Hasnaoui A. The clinical burden of allergic rhinitis in five Middle Eastern countries: results of the SNAPSHOT program. Allergy Asthma Clin Immunol 2018; 14: 63.

9. Alsowaidi S, Abdulle A, Shehab A, Zuberbier T, Bernsen R. Allergic rhinitis: prevalence and possible risk factors in a Gulf Arab population. Allergy 2010; 65: 208–212.

10. Alsowaidi S, Abdulle A, Bernsen R, Zuberbier T. Allergic rhinitis and asthma: a large cross-sectional study in the United Arab Emirates. Int. Arch. Allergy Immunol. 2010; 153: 274–279.

11. An S-Y. Analysis of various risk factors predisposing subjects to allergic rhinitis. Asian Pacific Journal of Allergy and Immunology 2015; .

12. Annesi-Maesano I, Didier A, Klossek M, Chanal I, Moreau D, Bousquet J. The score for allergic rhinitis (SFAR): a simple and valid assessment method in population studies. Allergy 2002; 57: 107–114.

13. Antonicelli L, Marchetti P, Accordini S, Bono R, Carosso A, Casali L, Cazzoletti L, Corsico A, Ferrari M, Fois A, Nicolini G, Olivieri M, Pirina P, Verlato G, Villani S, de Marco R. The Heterogeneity Hidden in Allergic Rhinitis and Its Impact on Co-Existing Asthma in Adults: A Population-Based Survey. Int. Arch. Allergy Immunol. 2015; 168: 205–212.

14. Bachert C, van Cauwenberge P, Olbrecht J, van Schoor J. Prevalence, classification and perception of allergic and nonallergic rhinitis in Belgium. Allergy 2006; 61: 693–698.

15. Bakke P, Gulsvik A, Eide GE. Hay fever, eczema and urticaria in southwest Norway.: Lifetime prevalences and association with sex, age, smoking habits, occupational airborne exposures and respiratory symptoms. Allergy 1990; 45: 515–522.

16. Baqueiro T, Pontes-de-carvalho L, Carvalho FM, Santos NM, Alcântara-Neves NM, Medical Student’s Group. Asthma and rhinitis symptoms in individuals from different socioeconomic levels in a Brazilian city. Allergy Asthma Proc 2007; 28: 362–367.

17. Başak O, Başak S, Gültekin B, Tekin N, Söylemez A. The prevalence of allergic rhinitis in adults in Aydin, Turkey. Rhinology 2006; 44: 283–287.

18. Bauchau V. Prevalence and rate of diagnosis of allergic rhinitis in Europe. European Respiratory Journal 2004; 24: 758–764.

19. Bergmann K-C, Heinrich J, Niemann H. Current status of allergy prevalence in Germany: Position paper of the Environmental Medicine Commission of the Robert Koch Institute. Allergo J Int 2016; 25: 6–10.

20. Billionnet C, Gay E, Kirchner S, Leynaert B, Annesi-Maesano I. Quantitative assessments of indoor air pollution and respiratory health in a population-based sample of French dwellings. Environ. Res. 2011; 111: 425–434.

21. Bjerg A, Ekerljung L, Middelveld R, Dahlén S-E, Forsberg B, Franklin K, Larsson K, Lötvall J, Ólafsdóttir IS, Torén K, Lundbäck B, Janson C. Increased Prevalence of Symptoms of Rhinitis but Not of Asthma between 1990 and 2008 in Swedish Adults: Comparisons of the ECRHS and GA2LEN Surveys. PLoS One 2011; 6.

22. Blackwell DL, Collins JG, Coles R. Summary health statistics for U.S. adults: National Health Interview Survey, 1997. Vital Health Stat 10 2002; : 1–109.

23. Blackwell DL, Lucas JW, Clarke TC. Summary health statistics for U.S. adults: national health interview survey, 2012. Vital Health Stat 10 2014; : 1–161.

24. Blomme K, Tomassen P, Lapeere H, Huvenne W, Bonny M, Acke F, Bachert C, Gevaert P. Prevalence of allergic sensitization versus allergic rhinitis symptoms in an unselected population. Int. Arch. Allergy Immunol. 2013; 160: 200–207.

25. Borna E, Nwaru BI, Bjerg A, Mincheva R, Rådinger M, Lundbäck B, Ekerljung L. Changes in the prevalence of asthma and respiratory symptoms in western Sweden between 2008 and 2016. Allergy 2019; .

26. Bousquet P-J, Leynaert B, Neukirch F, Sunyer J, Janson CM, Anto J, Jarvis D, Burney P. Geographical distribution of atopic rhinitis in the European Community Respiratory Health Survey I. Allergy 2008; 63: 1301–1309.

27. Broder I, Higgins MW, Mathews KP, Keller JB. Epidemiology of asthma and allergic rhinitis in a total community, Tecumseh, Michigan. 3. Second survey of the community. J. Allergy Clin. Immunol. 1974; 53: 127–138.

28. Bugiani M, Carosso A, Piccioni P, Migliore E, Corsico A, Ferrari M, de Marco R. Prevalence of allergic rhinitis in young adults in Italy. International Congress Series 2003; 1240: 497–501.

29. Bunjean K, Sukkasem K, Noppacroh N, Yamkaew N, Janthayanont D, Theerapancharern W, Chokkanchitchai S, Moungthong G. Prevalence of allergic rhinitis and types of sensitized allergen in adult at Wat Intaram community, Hua Raeu, Phra Nakhon Si Ayutthaya District, Phra Nakhon Si Ayutthaya Province, Thailand. J Med Assoc Thai 2012; 95 Suppl 5: S63-68.

30. Bunnag C, Jareoncharsri P, Voraprayoon S, Kongpatanakul S. Epidemiology of rhinitis in Thais: characteristics and risk factors. Asian Pac. J. Allergy Immunol. 2000; 18: 1–7.

31. Canuel M, Lebel G. Epidemiology of allergic rhinitis in Quebec: from a 2008 population-based survey. Chronic Dis Inj Can 2014; 34: 163–168.

32. Cardell L-O, Olsson P, Andersson M, Welin K-O, Svensson J, Tennvall GR, Hellgren J. TOTALL: high cost of allergic rhinitis—a national Swedish population-based questionnaire study. npj Primary Care Respiratory Medicine 2016; 26: 15082.

33. Cazzoletti L, Ferrari M, Olivieri M, Verlato G, Antonicelli L, Bono R, Casali L, Cerveri I, Marchetti P, Pirina P, Rossi A, Villani S, de Marco R. The gender, age and risk factor distribution differs in self-reported allergic and non-allergic rhinitis: a cross-sectional population-based study. Allergy Asthma Clin Immunol 2015; 11.

34. Cesaroni G, Badaloni C, Porta D, Forastiere F, Perucci CA. Comparison between various indices of exposure to traffic-related air pollution and their impact on respiratory health in adults. Occup Environ Med 2008; 65: 683–690.

35. Charpin D, Hughes B, Mallea M, Sutra JP, Balansard G, Vervloet D. Seasonal allergic symptoms and their relation to pollen exposure in south-east France. Clin. Exp. Allergy 1993; 23: 435–439.

36. Chia SE, Lim WK, Koh D. A prevalence study of chronic rhinitis among residents in Telok Blangah Town, Singapore. Ann. Acad. Med. Singap. 1994; 23: 358–362.

37. Cingi C, Songu M, Ural A, Annesi-Maesano I, Erdogmus N, Bal C, Kahya V, Koc EAO, Cakir BO, Selcuk A, Ozlugedik S, Onal K, Midilli R, Ecevit C, Pinar E, Akoglu E, Okuyucu S, Erkan AN. The Score For Allergic Rhinitis study in Turkey. Am J Rhinol Allergy 2011; 25: 333–337.

38. Cingi C, Topuz B, Songu M, Kara CO, Ural A, Yaz A, Yildirim M, Miman MC, Bal C. Prevalence of allergic rhinitis among the adult population in Turkey. Acta Otolaryngol. 2010; 130: 600–606.

39. de Marco R, Poli A, Ferrari M, Accordini S, Giammanco G, Bugiani M, Villani S, Ponzio M, Bono R, Carrozzi L, Cavallini R, Cazzoletti L, Dallari R, Ginesu F, Lauriola P, Mandrioli P, Perfetti L, Pignato S, Pirina P, Struzzo P, ISAYA study group. Italian Study on Asthma in Young Adults. The impact of climate and traffic-related NO2 on the prevalence of asthma and allergic rhinitis in Italy. Clin. Exp. Allergy 2002; 32: 1405–1412.

40. Dennis R, Caraballo L, García E, Caballero A, Aristizabal G, Córdoba H, Rodriguez MN, Rojas MX, Orduz C, Cardona R, Blanco A, Egea E, Verbel C, Cala LL. Asthma and other allergic conditions in Colombia: a study in 6 cities. Ann. Allergy Asthma Immunol. 2004; 93: 568–574.

41. Dennis RJ, Caraballo L, García E, Rojas MX, Rondon MA, Pérez A, Aristizabal G, Peñaranda A, Barragan AM, Ahumada V, Jimenez S. Prevalence of asthma and other allergic conditions in Colombia 2009-2010: a cross-sectional study. BMC Pulm Med 2012; 12: 17.

42. Derebery J, Meltzer E, Nathan RA, Stang PE, Campbell UB, Corrao M, Stanford R. Rhinitis symptoms and comorbidities in the United States: burden of rhinitis in America survey. Otolaryngol Head Neck Surg 2008; 139: 198–205.

43. Desalu OO, Salami AK, Iseh KR, Oluboyo PO. Prevalence of self reported allergic rhinitis and its relationship with asthma among adult Nigerians. J Investig Allergol Clin Immunol 2009; 19: 474–480.

44. Dotterud L, Odland J, Falk E. Atopic diseases among adults in the two geographically related arctic areas Nikel, Russia and Sør-Varanger, Norway: possible effects of indoor and outdoor air pollution. Journal of the European Academy of Dermatology and Venereology 2000; 14: 107–111.

45. Droste J, Kerkhof M, Demonchy J, Schouten J, Rijcken B. Association of skin test reactivity, specific IgE, total IgE, and eosinophils with nasal symptoms in a community-based population study+. Journal of Allergy and Clinical Immunology 1996; 97: 922–932.

46. ECRHS. Variations in the prevalence of respiratory symptoms, self-reported asthma attacks, and use of asthma medication in the European Community Respiratory Health Survey (ECRHS). European Respiratory Journal 1996; 9: 687–695.

47. Ekici A, Ekici M, Kocyigit P, Karlidag A. Prevalence of self-reported asthma in urban and rural areas of Turkey. J Asthma 2012; 49: 522–526.

48. El Kettani S, LotfiA B, Aichane A. [Prevalence of allergic rhinitis in a rural area of Settat, Morocco]. East. Mediterr. Health J. 2009; 15: 167–177.

49. Enright PL, Kronmal RA, Higgins MW, Schenker MB, Haponik EF. Prevalence and correlates of respiratory symptoms and disease in the elderly. Cardiovascular Health Study. Chest 1994; 106: 827–834.

50. Eriksson J, Ekerljung L, Pullerits T, Holmberg K, Rönmark E, Lötvall J, Lundbäck B. Prevalence of chronic nasal symptoms in West Sweden: risk factors and relation to self-reported allergic rhinitis and lower respiratory symptoms. Int. Arch. Allergy Immunol. 2011; 154: 155–163.

51. Eriksson J, Ekerljung L, Rönmark E, Dahlén B, Ahlstedt S, Dahlén S-E, Lundbäck B. Update of prevalence of self-reported allergic rhinitis and chronic nasal symptoms among adults in Sweden. Clin Respir J 2012; 6: 159–168.

52. Fazlollahi MR, Souzanch G, Nourizadeh M, Sabetkish N, Tazesh B, Entezari A, Pourpak Z. The Prevalence of Allergic Rhinitis and It’s Relationship With Second-Hand Tobacco Smoke Among Adults in Iran. Acta Med Iran 2017; 55: 712–717.

53. Filipiak B, Heinrich J, Nowak D, Wichmann HE. The distribution in specific IgE and the prevalence of allergic symptoms in 25-64-years old inhabitants of an eastern and a western German city--results from Augsburg and Erfurt. Eur J Epidemiol 2001; 17: 77–84.

54. Flatin M-C, Ade S, Hounkpatin S-H-R, Ametonou B, Vodouhe U-B, Adjibabi W. Symptoms of allergic rhinitis in Parakou, Benin: Prevalence, severity and associated factors. Eur Ann Otorhinolaryngol Head Neck Dis 2018; 135: 33–36.

55. Grønhøj Larsen C, Gyldenløve M, Linneberg A. Allergic rhinitis is often undiagnosed and untreated: results from a general population study of Danish adults. Clin Respir J 2013; 7: 354–358.

56. Ha J, Lee SW, Yon DK. 10-year trends and prevalence of asthma, allergic rhinitis, and atopic dermatitis among the Korean population, 2008-2017. Clin Exp Pediatr 2020; .

57. Håkansson K, von Buchwald C, Thomsen SF, Thyssen JP, Backer V, Linneberg A. Nonallergic rhinitis and its association with smoking and lower airway disease: A general population study. Am J Rhinol Allergy 2011; 25: 25–29.

58. Hannaford PC, Simpson JA, Bisset AF, Davis A, McKerrow W, Mills R. The prevalence of ear, nose and throat problems in the community: results from a national cross-sectional postal survey in Scotland. Family Practice 2005; 22: 227–233.

59. Hedman J, Kaprio J, Poussa T, Nieminen MM. Prevalence of asthma, aspirin intolerance, nasal polyposis and chronic obstructive pulmonary disease in a population-based study. Int J Epidemiol 1999; 28: 717–722.

60. Heinrich J, Nowak D, Wassmer G, Jörres R, Wjst M, Berger J, Magnussen H, Wichmann HE. Age-dependent differences in the prevalence of allergic rhinitis and atopic sensitization between an eastern and a western German city. Allergy 1998; 53: 89–93.

61. Heinrich J, Richter K, Magnussen H, Wichmann HE. Is the prevalence of atopic diseases in East and West Germany already converging? Eur. J. Epidemiol. 1998; 14: 239–245.

62. Heinrich J, Topp R, Gehring U, Thefeld W. Traffic at residential address, respiratory health, and atopy in adults: the National German Health Survey 1998. Environ. Res. 2005; 98: 240–249.

63. Hersoug L-G, Husemoen LLN, Thomsen SF, Sigsgaard T, Thuesen BH, Linneberg A. Association of indoor air pollution with rhinitis symptoms, atopy and nitric oxide levels in exhaled air. Int. Arch. Allergy Immunol. 2010; 153: 403–412.

64. Hopper JL, Jenkins MA, Carlin JB, Giles GG. Increase in the self-reported prevalence of asthma and hay fever in adults over the last generation: a matched parent-offspring study. Aust J Public Health 1995; 19: 120–124.

65. Hoppin JA, Jaramillo R, London SJ, Bertelsen RJ, Salo PM, Sandler DP, Zeldin DC. Phthalate Exposure and Allergy in the U.S. Population: Results from NHANES 2005–2006. Environ Health Perspect 2013; 121: 1129–1134.

66. Huang Y, Zhang Y, Zhang L. Prevalence of allergic and nonallergic rhinitis in a rural area of northern China based on sensitization to specific aeroallergens. Allergy Asthma Clin Immunol 2018; 14: 77.

67. Hwang C-Y, Chen Y-J, Lin M-W, Chen T-J, Chu S-Y, Chen C-C, Lee D-D, Chang Y-T, Wang W-J, Liu H-N. Prevalence of atopic dermatitis, allergic rhinitis and asthma in Taiwan: a national study 2000 to 2007. Acta Derm. Venereol. 2010; 90: 589–594.

68. Janson C, Johannessen A, Franklin K, Svanes C, Schiöler L, Malinovschi A, Gislason T, Benediktsdottir B, Schlünssen V, Jõgi R, Jarvis D, Lindberg E. Change in the prevalence asthma, rhinitis and respiratory symptom over a 20 year period: associations to year of birth, life style and sleep related symptoms. BMC Pulm Med [Internet] 2018 [cited 2019 Jul 30]; 18Available from: https://www.ncbi.nlm.nih.gov/pmc/articles/PMC6136212/.

69. Jarvis D, Newson R, Lotvall J, Hastan D, Tomassen P, Keil T, Gjomarkaj M, Forsberg B, Gunnbjornsdottir M, Minov J, Brozek G, Dahlen SE, Toskala E, Kowalski ML, Olze H, Howarth P, Krämer U, Baelum J, Loureiro C, Kasper L, Bousquet PJ, Bousquet J, Bachert C, Fokkens W, Burney P. Asthma in adults and its association with chronic rhinosinusitis: The GA2LEN survey in Europe. Allergy 2012; 67: 91–98.

70. Jõgi R, Janson C, Björnsson E, Boman G, Björkstén B. The prevalence of asthmatic respiratory symptoms among adults in Estonian and Swedish university cities. Allergy 1996; 51: 331–336.

71. Jones NS, Smith PA, Carney AS, Davis A. The prevalence of allergic rhinitis and nasal symptoms in Nottingham. Clin Otolaryngol Allied Sci 1998; 23: 547–554.

72. Jousilahti P, Haahtela T, Laatikainen T, Mäkelä M, Vartiainen E. Asthma and respiratory allergy prevalence is still increasing among Finnish young adults. Eur Respir J 2016; 47: 985–987.

73. Kakaje A, Alhalabi MM, Alyousbashi A, Hamid A, Hosam Aldeen O. Allergic Rhinitis and Its Epidemiological Distribution in Syria: A High Prevalence and Additional Risks in War Time. Biomed Res Int 2020; 2020: 7212037.

74. Katelaris CH, Lai CKW, Rhee C-S, Lee SH, Yun WD, Lim-Varona L, Quang VT, Hwang J, Singh H, Kim J, Boyle JM, Dhong HJ, Narayanan P, Vicente G, Blaiss M, Sacks R. Nasal allergies in the Asian-Pacific population: results from the Allergies in Asia-Pacific Survey. Am J Rhinol Allergy 2011; 25 Suppl 1: S3-15.

75. Kim DH, Han K, Kim SW. Relationship Between Allergic Rhinitis and Mental Health in the General Korean Adult Population. Allergy Asthma Immunol Res 2016; 8: 49–54.

76. Kim T-B, Kim Y-K, Chang Y-S, Kim S-H, Hong S-C, Jee Y-K, Cho S-H, Min K-U, Kim Y-Y. Association Between Sensitization to Outdoor Spider Mites and Clinical Manifestations of Asthma and Rhinitis in the General Population of Adults. J Korean Med Sci 2006; 21: 247–252.

77. Klossek J-M, Annesi-Maesano I, Pribil C, Didier A. Un tiers des adultes ont une rhinite allergique en France (enquête INSTANT). La Presse Médicale 2009; 38: 1220–1229.

78. Konno S, Hizawa N, Fukutomi Y, Taniguchi M, Kawagishi Y, Okada C, Tanimoto Y, Takahashi K, Akasawa A, Akiyama K, Nishimura M. The prevalence of rhinitis and its association with smoking and obesity in a nationwide survey of Japanese adults. Allergy 2012; 67: 653–660.

79. Kotaniemi J-T, Pallasaho P, Sovijärvi ARA, Laitinen LA, Lundbäck B. Respiratory symptoms and asthma in relation to cold climate, inhaled allergens, and irritants: a comparison between northern and southern Finland. J Asthma 2002; 39: 649–658.

80. Kurt E, Metintas S, Basyigit I, Bulut I, Coskun E, Dabak S, Deveci F, Fidan F, Kaynar H, Kunt Uzaslan E, Onbasi K, Ozkurt S, Pasaoglu Karakis G, Sahan S, Sahin U, Oguzulgen K, Yildiz F, Mungan D, Yorgancioglu A, Gemicioglu B, Fuat Kalyoncu A, PARFAIT Study of the Turkish Thoracic Society Asthma and Allergy Working Group. Prevalence and Risk Factors of Allergies in Turkey (PARFAIT): results of a multicentre cross-sectional study in adults. Eur. Respir. J. 2009; 33: 724–733.

81. Laatikainen T, von Hertzen L, Koskinen J-P, Mäkelä MJ, Jousilahti P, Kosunen TU, Vlasoff T, Ahlström M, Vartiainen E, Haahtela T. Allergy gap between Finnish and Russian Karelia on increase. Allergy 2011; 66: 886–892.

82. Lâm HT, Văn T Tng N, Ekerljung L, Rönmark E, Lundbäck B. Allergic rhinitis in northern vietnam: increased risk of urban living according to a large population survey. Clin Transl Allergy 2011; 1: 7.

83. Leth-Møller KB, Skaaby T, Linneberg A. Allergic rhinitis and allergic sensitisation are still increasing among Danish adults. Allergy 2019; .

84. Lethbridge-Çejku M, Rose D, Vickerie J. Summary health statistics for u.s. Adults: national health interview survey, 2004. Vital Health Stat 10 2006; : 1–164.

85. Lethbridge-Cejku M, Schiller JS, Bernadel L. Summary health statistics for U.S. adults: National Health Interview Survey, 2002. Vital Health Stat 10 2004; : 1–151.

86. Lethbridge-Çejku M, Vickerie J. Summary health statistics for u.s. Adults: national health interview survey, 2003. Vital Health Stat 10 2005; : 1–161.

87. Li C, Cheung C-L, Cheung TT, Samaranayake NR, Cheung BMY. Hay fever and hypertension in the US adult population. Clin. Exp. Hypertens. 2014; 36: 206–210.

88. Li CW, Chen DH, Zhong JT, Lin ZB, Peng H, Lu HG, Yang Y, Yin J, Li TY. Epidemiological characterization and risk factors of allergic rhinitis in the general population in Guangzhou City in china. PLoS ONE 2014; 9: e114950.

89. Lindgren A, Stroh E, Nihlén U, Montnémery P, Axmon A, Jakobsson K. Traffic exposure associated with allergic asthma and allergic rhinitis in adults. A cross-sectional study in southern Sweden. Int J Health Geogr 2009; 8: 25.

90. Linneberg A, Jørgensen T, Nielsen NH, Madsen F, Frølund L, Dirksen A. The prevalence of skin-test-positive allergic rhinitis in Danish adults: two cross-sectional surveys 8 years apart.The Copenhagen Allergy Study. Allergy 2000; 55: 767–772.

91. Linneberg A, Nielsen NH, Madsen F, Frølund L, Dirksen A, Jørgensen T. Increasing prevalence of allergic rhinitis symptoms in an adult Danish population. Allergy 1999; 54: 1194–1198.

92. Linneberg A, Nielsen NH, Madsen F, Frølund L, Dirksen A, Jørgensen T. Increasing prevalence of specific IgE to aeroallergens in an adult population: Two cross-sectional surveys 8 years apart. Journal of Allergy and Clinical Immunology 2000; 106: 247–252.

93. Lötvall J, Ekerljung L, Rönmark EP, Wennergren G, Lindén A, Rönmark E, Torén K, Lundbäck B. West Sweden Asthma Study: prevalence trends over the last 18 years argues no recent increase in asthma. Respir. Res. 2009; 10: 94.

94. Lucas JW, Schiller JS, Benson V. Summary health statistics for U.S. adults: National Health Interview Survey, 2001. Vital Health Stat 10 2004; : 1–134.

95. Ma T, Wang X, Zhuang Y, Shi H, Ning H, Lan T, Zhang T, Kang Z, SiQin B, Yang B, Bao X, Yan W, Lei T, Wang D, Shan G, Zhang B, Wang X, Zhang L. Prevalence and risk factors for allergic rhinitis in adults and children living in different grassland regions of Inner Mongolia. Allergy 2019; .

96. Mahboub B, Al-Hammadi S, Prakash VP, Sulaiman N, Blaiss MS, Redha AA, Vats DM. Prevalence and triggers of allergic rhinitis in the United Arab Emirates. World Allergy Organ J 2014; 7: 19.

97. Marco R de, Cappa V, Accordini S, Rava M, Antonicelli L, Bortolami O, Braggion M, Bugiani M, Casali L, Cazzoletti L, Cerveri I, Fois AG, Girardi P, Locatelli F, Marcon A, Marinoni A, Panico MG, Pirina P, Villani S, Zanolin ME, Verlato G. Trends in the prevalence of asthma and allergic rhinitis in Italy between 1991 and 2010. European Respiratory Journal 2012; 39: 883–892.

98. Marshall AH, Owen V, Jones NS. More siblings, less hay fever: more evidence. Clin Otolaryngol Allied Sci 2002; 27: 352–358.

99. Meltzer EO, Blaiss MS, Naclerio RM, Stoloff SW, Derebery MJ, Nelson HS, Boyle JM, Wingertzahn MA. Burden of allergic rhinitis: allergies in America, Latin America, and Asia-Pacific adult surveys. Allergy Asthma Proc 2012; 33 Suppl 1: S113-141.

100. Milenković B, Mitić-Milikić M, Rebić P, Vukcević M, Dudvarski-Ilić A, Nagorni-Obradović L, Lazić Z, Bosnjak-Petrović V. Asthma and chronic bronchitis symptoms among adult population of Belgrade. Srp Arh Celok Lek 2011; 139: 149–154.

101. Min YG, Jung HW, Kim HS, Park SK, Yoo KY. Prevalence and risk factors for perennial allergic rhinitis in Korea: results of a nationwide survey. Clin Otolaryngol Allied Sci 1997; 22: 139–144.

102. Montnémery P, Popovic M, Andersson M, Greiff L, Nyberg P, Löfdahl CG, Svensson C, Persson CGA. Influence of heavy traffic, city dwelling and socio-economic status on nasal symptoms assessed in a postal population survey. Respir Med 2003; 97: 970–977.

103. Montnémery P, Svensson C, Adelroth E, Löfdahl CG, Andersson M, Greiff L, Persson CG. Prevalence of nasal symptoms and their relation to self-reported asthma and chronic bronchitis/emphysema. Eur Respir J 2001; 17: 596–603.

104. Morais-Almeida M, Pite H, Pereira AM, Todo-Bom A, Nunes C, Bousquet J, Fonseca J. Prevalence and classification of rhinitis in the elderly: a nationwide survey in Portugal. Allergy 2013; 68: 1150–1157.

105. Morgan BW, Siddharthan T, Grigsby MR, Pollard SL, Kalyesubula R, Wise RA, Kirenga B, Checkley W. Asthma and Allergic Disorders in Uganda: A Population-Based Study Across Urban and Rural Settings. J Allergy Clin Immunol Pract 2018; 6: 1580-1587.e2.

106. Myong J-P, Kim H, Lee K, Chang S. Time Trends of Allergic Rhinitis and Effects of Residence on Allergic Rhinitis in Korea From 1998 Through 2007–2009. Asian Nursing Research 2012; 6: 102–106.

107. Nam J-S, Hwang C-S, Hong M-P, Kim K-S. Prevalence and clinical characteristics of allergic rhinitis in the elderly Korean population. Eur Arch Otorhinolaryngol 2020; .

108. Nathan RA, Meltzer EO, Derebery J, Campbell UB, Stang PE, Corrao MA, Allen G, Stanford R. The prevalence of nasal symptoms attributed to allergies in the United States: Findings from the burden of rhinitis in an America survey. allergy asthma proc 2008; 29: 600–608.

109. Neffen H, Mello JF, Sole D, Naspitz CK, Dodero AE, Garza HL, Guerra EN, Baez-Loyola C, Boyle JM, Wingertzahn MA. Nasal allergies in the Latin American population: results from the Allergies in Latin America survey. Allergy Asthma Proc 2010; 31 Suppl 1: S9-27.

110. Ng TP, Tan WC. Epidemiology of chronic (perennial) rhinitis in Singapore: prevalence estimates, demographic variation and clinical allergic presentation. Ann. Acad. Med. Singap. 1994; 23: 83–88.

111. Ng TP, Tan WC. Epidemiology of allergic rhinitis and its associated risk factors in Singapore. Int J Epidemiol 1994; 23: 553–558.

112. Nihlén U, Greiff L, Montnémery P, Löfdahl C-G, Johannisson A, Persson C, Andersson M. Incidence and remission of self-reported allergic rhinitis symptoms in adults. Allergy 2006; 61: 1299–1304.

113. Nowak D, Heinrich J, Jörres R, Wassmer G, Berger J, Beck E, Boczor S, Claussen M, Wichmann HE, Magnussen H. Prevalence of respiratory symptoms, bronchial hyperresponsiveness and atopy among adults: west and east Germany. Eur Respir J 1996; 9: 2541–2552.

114. Nyembue TD, Jorissen M, Hellings PW, Muyunga C, Kayembe JM. Prevalence and determinants of allergic diseases in a Congolese population. International Forum of Allergy & Rhinology 2012; 2: 285–293.

115. Oliveira TB, Persigo ALK, Ferrazza CC, Ferreira ENN, Veiga ABG. Prevalence of asthma, allergic rhinitis and pollinosis in a city of Brazil: A monitoring study. Allergol Immunopathol (Madr) 2020; .

116. Olivieri M, Verlato G, Corsico A, Lo Cascio V, Bugiani M, Marinoni A, de Marco R, Italian European Community Respiratory Health Survey group. Prevalence and features of allergic rhinitis in Italy. Allergy 2002; 57: 600–606.

117. Olsson P, Berglind N, Bellander T, Stjärne P. Prevalence of self-reported allergic and non-allergic rhinitis symptoms in Stockholm: relation to age, gender, olfactory sense and smoking. Acta Otolaryngol. 2003; 123: 75–80.

118. Ozoh OB, Aderibigbe SA, Ayuk AC, Desalu OO, Oridota OE, Olufemi O, Egbagbe E, Babashani M, Shopeyin A, Ukwaja K, Dede SK. The prevalence of asthma and allergic rhinitis in Nigeria: A nationwide survey among children, adolescents and adults. PLOS ONE 2019; 14: e0222281.

119. Pakkasela J, Ilmarinen P, Honkamäki J, Tuomisto LE, Andersén H, Piirilä P, Hisinger-Mölkänen H, Sovijärvi A, Backman H, Lundbäck B, Rönmark E, Kankaanranta H, Lehtimäki L. Age-specific incidence of allergic and non-allergic asthma. BMC Pulm Med 2020; 20: 9.

120. Pallasaho P, Kainu A, Juusela M, Meren M, Sovijärvi A. High prevalence of rhinitis symptoms without allergic sensitization in Estonia and Finland. Eur Clin Respir J 2015; 2.

121. Pariente PD, LePen C, Los F, Bousquet J. Quality-of-Life Outcomes and the Use of Antihistamines in a French National Population-Based Sample of Patients with Perennial Rhinitis: PharmacoEconomics 1997; 12: 585–595.

122. Park HJ, Kim EJ, Yoon D, Lee JK, Chang WS, Lim YM, Park JW, Lee JS. Prevalence of Self-reported Allergic Diseases and IgE Levels: A 2010 KNHANES Analysis. Allergy Asthma Immunol Res 2017; 9: 329–339.

123. Park S, Jung PK, Choi M, Seok H, Kim H, Oh S-S, Koh S-B. Association between occupational clusters and allergic rhinitis in the Korean population: analysis of the Korean National Health and Nutrition Examination Survey data. J Occup Health 2018; 60: 312–319.

124. Peat JK, Haby M, Spijker J, Berry G, Woolcock AJ. Prevalence of asthma in adults in Busselton, Western Australia. BMJ 1992; 305: 1326–1329.

125. Pefura-Yone EW, Kengne AP, Balkissou AD, Boulleys-Nana JR, Efe-de-Melingui NR, Ndjeutcheu-Moualeu PI, Mbele-Onana CL, Kenmegne-Noumsi EC, Kolontchang-Yomi BL, Theubo-Kamgang BJ, Ebouki ER, Djuikam-Kamga CK, Magne-Fotso CG, Amougou F, Mboumtou L, Ngo-Yonga M, Petchou-Talla EL, Afane-Ze E, Kuaban C, Research Group for Respiratory Disease in Cameroon (RGRDC). Prevalence of asthma and allergic rhinitis among adults in Yaounde, Cameroon. PLoS ONE 2015; 10: e0123099.

126. Pleis JR, Coles R. Summary health statistics for U.S. adults: National Health Interview Survey, 1998. Vital Health Stat 10 2002; : 1–113.

127. Pleis JR, Coles R. Summary health statistics for U.S. adults: National Health Interview Survey, 1999. Vital Health Stat 10 2003; : 1–137.

128. Pleis JR, Lethbridge-Cejku M. Summary health statistics for U.S. adults: National Health Interview Survey, 2005. Vital Health Stat 10 2006; : 1–153.

129. Pleis JR, Lethbridge-Cejku M. Summary health statistics for U.S. adults: National Health Interview Survey, 2006. Vital Health Stat 10 2007; : 1–153.

130. Pleis JR, Lucas JW. Summary health statistics for U.S. adults: National Health Interview Survey, 2007. Vital Health Stat 10 2009; : 1–159.

131. Pleis JR, Lucas JW, Ward BW. Summary health statistics for U.S. adults: National Health Interview Survey, 2008. Vital Health Stat 10 2009; : 1–157.

132. Pleis JR, Schiller JS, Benson V. Summary health statistics for U.S. adults: National Health Interview Survey, 2000. Vital Health Stat 10 2003; : 1–132.

133. Pleis JR, Ward BW, Lucas JW. Summary health statistics for U.S. adults: National Health Interview Survey, 2009. Vital Health Stat 10 2010; : 1–207.

134. Quercia O, Incorvaia C, Puccinelli P, Scurati S, Emiliani F, Frati F, Stefanini GF. Prevalence of allergic disorders in Italy: the Cotignola population study. Eur Ann Allergy Clin Immunol 2012; 44: 5–11.

135. Rahimi-Rad MH, Gaderi-Pakdel F, Salari-Lak S. Smoking and asthma in 20-44-year-old adults in Urmia, Islamic Republic of Iran. East Mediterr Health J 2008; 14: 6–16.

136. Rennie DC, Karunanayake CP, Chen Y, Lawson JA, Hagel L, Senthilselvan A, Pahwa P, Dosman JA, Saskatchewan Rural Cohort Study Group. Early farm residency and prevalence of asthma and hay fever in adults. J Asthma 2016; 53: 2–10.

137. Rhee C-S, Wee JH, Ahn J-C, Lee WH, Tan KL, Ahn S, Lee JH, Lee C-H, Cho Y-S, Park KH, Lee KH, Kim K-S, Lee A, Kim J-W. Prevalence, risk factors and comorbidities of allergic rhinitis in South Korea: The Fifth Korea National Health and Nutrition Examination Survey. Am J Rhinol Allergy 2014; 28: e107-114.

138. Richards S, Thornhill D, Roberts H, Harries U. How many people think they have hay fever, and what they do about it. Br J Gen Pract 1992; 42: 284–286.

139. Rodríguez Portal JA, Alvarez Gutiérrez FJ, Segado Soriano A, Soto Campos G, Capote Gil F, Castillo Gómez J. [An analysis of the prevalence of respiratory symptomatology in the general population]. Arch Bronconeumol 1995; 31: 162–168.

140. Rönmark EP, Ekerljung L, Mincheva R, Sjölander S, Hagstad S, Wennergren G, Rönmark E, Lötvall J, Lundbäck B. Different risk factor patterns for adult asthma, rhinitis and eczema: results from West Sweden Asthma Study. Clin Transl Allergy 2016; 6.

141. Roxbury CR, Qiu M, Shargorodsky J, Lin SY. Association between allergic rhinitis and poor sleep parameters in U.S. adults: Sleep parameters and allergic rhinitis. Int Forum Allergy Rhinol. 2018; 8: 1098–1106.

142. Sa-Sousa A, Morais-Almeida M, Azevedo LF, Carvalho R, Jacinto T, Todo-Bom A, Loureiro C, Bugalho-Almeida A, Bousquet J, Fonseca JA. Prevalence of asthma in Portugal - The Portuguese National Asthma Survey. Clin Transl Allergy 2012; 2: 15.

143. Sakar A, Yorgancioglu A, Dinc G, Yuksel H, Celik P, Dagyildizi L, Coskun E, Kaya E, Ozyurt B, Ozcan C. The prevalence of asthma and allergic symptoms in Manisa, Turkey (A western city from a country bridging Asia and Europe). Asian Pac. J. Allergy Immunol. 2006; 24: 17–25.

144. Sakashita M, Hirota T, Harada M, Nakamichi R, Tsunoda T, Osawa Y, Kojima A, Okamoto M, Suzuki D, Kubo S, Imoto Y, Nakamura Y, Tamari M, Fujieda S. Prevalence of allergic rhinitis and sensitization to common aeroallergens in a Japanese population. Int. Arch. Allergy Immunol. 2010; 151: 255–261.

145. Salo PM, Calatroni A, Gergen PJ, Hoppin JA, Sever ML, Jaramillo R, Arbes SJ, Zeldin DC. Allergy-related outcomes in relation to serum IgE: Results from the National Health and Nutrition Examination Survey 2005–2006. J Allergy Clin Immunol 2011; 127: 1226-1235.e7.

146. Samoliński B, Sybilski AJ, Raciborski F, Tomaszewska A, Samel-Kowalik P, Walkiewicz A, Lusawa A, Borowicz J, Gutowska-Slesik J, Trzpil L, Marszałkowska J, Jakubik N, Krzych E, Komorowski J, Lipiec A, Gotlib T, Samolińska-Zawisza U, Hałat Z. Prevalence of rhinitis in Polish population according to the ECAP (Epidemiology of Allergic Disorders in Poland) study. Otolaryngol Pol 2009; 63: 324–330.

147. Schiller JS, Lucas JW, Peregoy JA. Summary health statistics for u.s. Adults: national health interview survey, 2011. Vital Health Stat 10 2012; : 1–218.

148. Schiller JS, Lucas JW, Ward BW, Peregoy JA. Summary health statistics for U.S. adults: National Health Interview Survey, 2010. Vital Health Stat 10 2012; : 1–207.

149. Shaaban R, Zureik M, Soussan D, Neukirch C, Heinrich J, Sunyer J, Wjst M, Cerveri I, Pin I, Bousquet J, Jarvis D, Burney PG, Neukirch F, Leynaert B. Rhinitis and onset of asthma: a longitudinal population-based study. Lancet 2008; 372: 1049–1057.

150. Shahar E, Lorber M. Prevalence of self-reported allergic conditions in an adult population in Israel. Isr. Med. Assoc. J. 2001; 3: 190–193.

151. Shargorodsky J, Garcia-Esquinas E, Galán I, Navas-Acien A, Lin SY. Allergic Sensitization, Rhinitis and Tobacco Smoke Exposure in US Adults. PLoS ONE 2015; 10: e0131957.

152. Shen J, Ke X, Hong S, Zeng Q, Liang C, Li T, Tang A. Epidemiological features of allergic rhinitis in four major cities in Western China. J. Huazhong Univ. Sci. Technol. Med. Sci. 2011; 31: 433.

153. SHOKOUHI SHOORMASTI R, POURPAK Z, FAZLOLLAHI MR, KAZEMNEJAD A, NADALI F, EBADI Z, TAYEBI B, MOSLEMI M, KARIMI A, VALMOHAMMADI S, NAZEMI AM, MARI A, MOIN M. The Prevalence of Allergic Rhinitis, Allergic Conjunctivitis, Atopic Dermatitis and Asthma among Adults of Tehran. Iran J Public Health 2018; 47: 1749–1755.

154. Sibbald B, Rink E. Labelling of rhinitis and hayfever by doctors. Thorax 1991; 46: 378–381.

155. Sichletidis L, Tsiotsios I, Gavriilidis A, Chloros D, Kottakis I, Daskalopoulou E, Konstantinidis T. Prevalence of chronic obstructive pulmonary disease and rhinitis in northern Greece. Respiration 2005; 72: 270–277.

156. Singh K, Axelrod S, Bielory L. The epidemiology of ocular and nasal allergy in the United States, 1988-1994. Journal of Allergy and Clinical Immunology 2010; 126: 778-783.e6.

157. Sinha B, Vibha, Singla R, Chowdhury R. Allergic Rhinitis: A neglected disease - A community based assessment among adults in Delhi. J Postgrad Med 2015; 61: 169–175.

158. Smith-Sivertsen T, Tchachtchine V, Lund E. Atopy in Norwegian and Russian adults: a population-based study from the common border area. Allergy 2003; 58: 357–362.

159. Song W-J, Kim M-Y, Jo E-J, Kim M-H, Kim T-H, Kim S-H, Kim K-W, Cho S-H, Min K-U, Chang Y-S. Rhinitis in a community elderly population: relationships with age, atopy, and asthma. Ann. Allergy Asthma Immunol. 2013; 111: 347–351.

160. Song W-J, Sohn K-H, Kang M-G, Park H-K, Kim M-Y, Kim S-H, Lim MK, Choi M-H, Kim KW, Cho S-H, Min K-U, Chang Y-S. Urban-rural differences in the prevalence of allergen sensitization and self-reported rhinitis in the elderly population. Ann. Allergy Asthma Immunol. 2015; 114: 455–461.

161. Sonia T, Meriem M, Yacine O, Nozha BS, Nadia M, Bechir L, Jalloul D, Jouda C, Majed B. Prevalence of asthma and rhinitis in a Tunisian population. Clin Respir J 2018; 12: 608–615.

162. Sonomjamts M, Dashdemberel S, Logii N, Nakae K, Chigusa Y, Ohhira S, Ito C, Sagara H, Makino S. Prevalence of asthma and allergic rhinitis among adult population in Ulaanbaatar, Mongolia. Asia Pac Allergy 2014; 4: 25–31.

163. Sozańska B, Błaszczyk M, Pearce N, Cullinan P. Atopy and allergic respiratory disease in rural Poland before and after accession to the European Union. J. Allergy Clin. Immunol. 2014; 133: 1347–1353.

164. Todo-Bom A, Loureiro C, Almeida MM, Nunes C, Delgado L, Castel-Branco G, Bousquet J. Epidemiology of rhinitis in Portugal: evaluation of the intermittent and the persistent types. Allergy 2007; 62: 1038–1043.

165. Tschopp JM, Sistek D, Schindler C, Leuenberger P, Perruchoud AP, Wüthrich B, Brutsche M, Zellweger JP, Karrer W, Brändli O, SAPALDIA team. Current allergic asthma and rhinitis: diagnostic efficiency of three commonly used atopic markers (IgE, skin prick tests, and Phadiatop®): Results from 8329 randomized adults from the SAPALDIA study. Allergy 1998; 53: 608–613.

166. Turkeltaub PC, Gergen PJ. Prevalence of upper and lower respiratory conditions in the US population by social and environmental factors: data from the second National Health and Nutrition Examination Survey, 1976 to 1980 (NHANES II). Ann Allergy 1991; 67: 147–154.

167. Upperman CR, Parker JD, Akinbami LJ, Jiang C, He X, Murtugudde R, Curriero FC, Ziska L, Sapkota A. Exposure to Extreme Heat Events Is Associated with Increased Hay Fever Prevalence among Nationally Representative Sample of US Adults: 1997-2013. The Journal of Allergy and Clinical Immunology: In Practice 2017; 5: 435-441.e2.

168. Upton MN, McConnachie A, McSharry C, Hart CL, Smith GD, Gillis CR, Watt GC. Intergenerational 20 year trends in the prevalence of asthma and hay fever in adults: the Midspan family study surveys of parents and offspring. BMJ 2000; 321: 88–92.

169. Verlato G, Corsico A, Villani S, Cerveri I, Migliore E, Accordini S, Carolei A, Piccioni P, Bugiani M, Lo Cascio V, Marinoni A, Poli A, de Marco R. Is the prevalence of adult asthma and allergic rhinitis still increasing? Results of an Italian study. J. Allergy Clin. Immunol. 2003; 111: 1232–1238.

170. Vervloet D, Haddi E, Tafforeau M, Lanteaume A, Kulling G, Charpin D. Reliability of respiratory symptoms to diagnose atopy. Clin Exp Allergy 1991; 21: 733–737.

171. Viegi G, Paoletti P, Carrozzi L, Vellutini M, Diviggiano E, Di Pede C, Pistelli G, Giutini G, Lebowitz MD. Prevalence rates of respiratory symptoms in Italian general population samples exposed to different levels of air pollution. Environ. Health Perspect. 1991; 94: 95–99.

172. Von Linstow M-L, Porsbjerg C, Ulrik CS, Nepper-Christensen S, Backer V. Prevalence and predictors of atopy among young Danish adults. Clin. Exp. Allergy 2002; 32: 520–525.

173. Wang D-Y, Niti M, Smith JD, Yeoh KH, Ng TP. Rhinitis: do diagnostic criteria affect the prevalence and treatment? Allergy 2002; 57: 150–154.

174. Wang J, Engvall K, Smedje G, Norbäck D. Rhinitis, asthma and respiratory infections among adults in relation to the home environment in multi-family buildings in Sweden. PLoS ONE 2014; 9: e105125.

175. Wang XD, Zheng M, Lou HF, Wang CS, Zhang Y, Bo MY, Ge SQ, Zhang N, Zhang L, Bachert C. An increased prevalence of self‐reported allergic rhinitis in major Chinese cities from 2005 to 2011. Allergy 2016; 71: 1170–1180.

176. Wang X-Y, Ma T-T, Wang X-Y, Zhuang Y, Wang X-D, Ning H-Y, Shi H-Y, Yu R-L, Yan D, Huang H-D, Bai Y-F, Shan G-L, Zhang B, Song Q-K, Zhang Y-F, Zhang T-J, Jia D-Z, Liu X-L, Kang Z-X, Yan W-J, Yang B-T, Bao X-Z, Sun S-H, Zhang F-F, Yu W-H, Bai C-L, Wei T, Yang T, Ma T-Q, Wu X-B, et al. Prevalence of pollen-induced allergic rhinitis with high pollen exposure in grasslands of northern China. Allergy 2018; 73: 1232–1243.

177. Warm K, Hedman L, Lindberg A, Lötvall J, Lundbäck B, Rönmark E. Allergic sensitization is age-dependently associated with rhinitis, but less so with asthma. J. Allergy Clin. Immunol. 2015; 136: 1559-1565.e2.

178. Wolkewitz M, Rothenbacher D, Löw M, Stegmaier C, Ziegler H, Radulescu M, Brenner H, Diepgen TL. Lifetime prevalence of self-reported atopic diseases in a population-based sample of elderly subjects: results of the ESTHER study. Br. J. Dermatol. 2007; 156: 693–697.

179. Woods RK, Walters EH, Wharton C, Watson N, Abramson M. The rising prevalence of asthma in young Melbourne adults is associated with improvement in treatment. Ann Allergy Asthma Immunol 2001; 87: 117–123.

180. Wüthrich B, Schindler C, Leuenberger P, Ackermann-Liebrich U. Prevalence of Atopy and Pollinosis in the Adult Population of Switzerland (SAPALDIA Study). International Archives of Allergy and Immunology 1995; 106: 149–156.

181. Wüthrich B, Schmid-Grendelmeier P, Schindler C, Imboden M, Bircher A, Zemp E, Probst-Hensch N, Team and the S. Prevalence of Atopy and Respiratory Allergic Diseases in the Elderly SAPALDIA Population. IAA 2013; 162: 143–148.

182. Zanolin ME, Pattaro C, Corsico A, Bugiani M, Carrozzi L, Casali L, Dallari R, Ferrari M, Marinoni A, Migliore E, Olivieri M, Pirina P, Verlato G, Villani S, Marco R, ISAYA Study Group. The role of climate on the geographic variability of asthma, allergic rhinitis and respiratory symptoms: results from the Italian study of asthma in young adults. Allergy 2004; 59: 306–314.

183. Zhang L, Han D, Huang D, Wu Y, Dong Z, Xu G, Kong W, Bachert C. Prevalence of self-reported allergic rhinitis in eleven major cities in china. Int. Arch. Allergy Immunol. 2009; 149: 47–57.

184. Zheng M, Wang X, Bo M, Wang K, Zhao Y, He F, Cao F, Zhang L, Bachert C. Prevalence of Allergic Rhinitis Among Adults in Urban and Rural Areas of China: A Population-Based Cross-Sectional Survey. Allergy Asthma Immunol Res 2015; 7: 148–157.

185. NHIS - Tables of Summary Health Statistics [Internet]. 2021 [cited 2021 Jul 19].Available from: https://www.cdc.gov/nchs/nhis/shs/tables.htm.

186. NHANES Questionnaires, Datasets, and Related Documentation [Internet]. [cited 2021 Jul 19].Available from: https://wwwn.cdc.gov/nchs/nhanes/continuousnhanes/default.aspx?BeginYear=2005.

187. NHANES Questionnaires, Datasets, and Related Documentation [Internet]. [cited 2021 Jul 19].Available from: https://wwwn.cdc.gov/nchs/nhanes/continuousnhanes/default.aspx?BeginYear=2007.

188. NHANES Questionnaires, Datasets, and Related Documentation [Internet]. [cited 2021 Jul 19].Available from: https://wwwn.cdc.gov/nchs/nhanes/continuousnhanes/default.aspx?BeginYear=2009.

189. NHANES Questionnaires, Datasets, and Related Documentation [Internet]. [cited 2021 Jul 19].Available from: https://wwwn.cdc.gov/nchs/nhanes/continuousnhanes/default.aspx?BeginYear=2011.

**Tables**

Table S1: Prevalences of rhinitis according to the different definitions (sensitivity analysis)

|  | **n** | **Mean** | **Med** | **Sd** | **CI_95%_** | | **Min** | **Max** |
| --- | --- | --- | --- | --- | --- | --- | --- | --- |
| **All definitions** | | | | | | | | |
| **Unspecified rhinitis** | 27 | 31.5% | 32.4% | 12.4% | (26.6%, | 36.4%) | 10.4% | 54.1% |
| **AR** | 63 | 19.1% | 17.1% | 9.6% | (16.7%, | 21.5%) | 1.00% | 44.2% |
| **NAR** | 3 | 15.5% | 16.4% | 8.54% |  |  | 6.49% | 23.5% |
| **Symptoms-based definitions** | | | | | | | | |
| **Unspecified rhinitis** | |  |  |  |  |  |  |  |
| Ever | 9 | 30.4% | 31.3% | 13.3% | (20.1%, | 40.6%) | 11.4% | 50.2% |
| Current | 17 | 33.1% | 34.6% | 11.8% | (27.0%, | 39.2%) | 10.40% | 54.1% |
| **AR** |  |  |  |  |  |  |  |  |
| Ever | 8 | 25.1% | 24.7% | 8.6% | (17.9%, | 32.4%) | 14.5% | 41.6% |
| Current | 18 | 22.9% | 25.5% | 10.7% | (17.6%, | 28.2%) | 3.6% | 37.7% |
| **NAR** |  |  |  |  |  |  |  |  |
| Ever | 0 |  |  |  |  |  |  |  |
| Current | 2 | 11.45% |  | 7.01% |  |  | 6.49% | 16.40% |
| **Doctor diagnosis-based definitions** | | | | | | | | |
| **Unspecified rhinitis** | |  |  |  |  |  |  |  |
| **Ever** | 1 | 14.0% |  |  |  |  |  |  |
| Current | 0 |  |  |  |  |  |  |  |
| **AR** |  |  |  |  |  |  |  |  |
| Ever | 24 | 14.6% | 13.4% | 7.00% | (11.6%, | 17.5%) | 1.00% | 30.6% |
| Current | 1 | 11.4% |  |  |  |  |  |  |
| **IgE/SPT-based definitions** | | | | | | | | |
| **AR** |  |  |  |  |  |  |  |  |
| Ever | 0 |  |  |  |  |  |  |  |
| Current | 12 | 18.9% | 17.7% | 9.53% | (12.9%, | 25.0%) | 6.50% | 44.2% |
| **NAR** |  |  |  |  |  |  |  |  |
| Ever | 0 |  |  |  |  |  |  |  |
| Current | 1 | 23.5% |  |  |  |  |  |  |

AR: Allergic Rhinitis; CI: Confidence Interval; Current: combination of point and period prevalences; Ever: Lifetime prevalences; Max: highest reported prevalence; Med: Median; Min: lowest reported prevalence; n: number of reported prevalences; NAR: Non-Allergic Rhinitis; sd: standard deviation.

Analyses after exclusion of: 1) definitions based on: a) questions that did not explicitly exclude common cold or the flu; b) questions that included other allergic diseases such as conjunctivitis or eczema; c) questions that were limited to the time of the survey; d) questions that were not specific of a timeframe and e) questions that focused solely on specific sub-phenotypes of rhinitis, such as rhino-conjunctivitis, seasonal AR, or peri-annual rhinitis (as prevalences of those sub-phenotypes reflect only a part of the prevalence of AR, NAR or unspecified rhinitis),

2) prevalences calculated on populations: a) that mixed adults and children (i.e. under the age of 16), and b) including only elder (i.e. above the age of 60).

Table S2: Prevalences of rhinitis in different countries

|  | **Unspecified rhinitis** | | | | |  | **AR** | | | | |  | **NAR** | | | | |
| --- | --- | --- | --- | --- | --- | --- | --- | --- | --- | --- | --- | --- | --- | --- | --- | --- | --- |
| **Country** | **n** | **Med** | **Mean** | **Min** | **Max** |  | **n** | **Med** | **Mean** | **Min** | **Max** |  | **n** | **Med** | **Mean** | **Min** | **Max** |
| **Africa** | **9** | **13.4%** | **21.3%** | **10.4%** | **37.8%** |  | **3** | **9.5%** | **12.0%** | **3.6%** | **22.8%** |  |  |  |  |  |  |
| Algeria |  |  |  |  |  |  | 1 | 9.5% | 9.5% | 9.5% | 9.5% |  |  |  |  |  |  |
| Benin | 1 | 35.7% | 35.7% | 35.7% | 35.7% |  |  |  |  |  |  |  |  |  |  |  |  |
| Cameroon | 2 | 10.9% | 10.9% | 10.4% | 11.4% |  |  |  |  |  |  |  |  |  |  |  |  |
| Congo | 1 | 30.8% | 30.8% | 30.8% | 30.8% |  |  |  |  |  |  |  |  |  |  |  |  |
| Egypt | 1 | 11.0% | 11.0% | 11.0% | 11.0% |  | 1 | 3.6% | 3.6% | 3.6% | 3.6% |  |  |  |  |  |  |
| Morocco | 1 | 37.8% | 37.8% | 37.8% | 37.8% |  |  |  |  |  |  |  |  |  |  |  |  |
| Nigeria | 1 | 29.6% | 29.6% | 29.6% | 29.6% |  | 1 | 22.8% | 22.8% | 22.8% | 22.8% |  |  |  |  |  |  |
| Tunisia | 1 | 13.4% | 13.4% | 13.4% | 13.4% |  |  |  |  |  |  |  |  |  |  |  |  |
| Uganda | 1 | 11.9% | 11.9% | 11.9% | 11.9% |  |  |  |  |  |  |  |  |  |  |  |  |
| **America** | **13** | **35.4%** | **36.9%** | **14.0%** | **63.3%** |  | **56** | **8.9%** | **14.1%** | **3.5%** | **54.5%** |  |  |  |  |  |  |
| Argentina |  |  |  |  |  |  | 1 | 3.5% | 3.5% | 3.5% | 3.5% |  |  |  |  |  |  |
| Brazil | 3 | 50.5% | 46.6% | 26.0% | 63.3% |  | 3 | 43.3% | 34.7% | 8.8% | 52.0% |  |  |  |  |  |  |
| Canada |  |  |  |  |  |  | 4 | 14.7% | 13.0% | 5.7% | 17.0% |  |  |  |  |  |  |
| Chile |  |  |  |  |  |  | 1 | 9.8% | 9.8% | 9.8% | 9.8% |  |  |  |  |  |  |
| Colombia | 2 | 35.0% | 35.0% | 32.0% | 38.0% |  | 2 | 14.8% | 14.8% | 7.0% | 22.6% |  |  |  |  |  |  |
| Ecuador |  |  |  |  |  |  | 1 | 6.4% | 6.4% | 6.4% | 6.4% |  |  |  |  |  |  |
| Mexico |  |  |  |  |  |  | 1 | 6.3% | 6.3% | 6.3% | 6.3% |  |  |  |  |  |  |
| Peru |  |  |  |  |  |  | 1 | 11.6% | 11.6% | 11.6% | 11.6% |  |  |  |  |  |  |
| United States | 8 | 34.8% | 33.8% | 14.0% | 46.2% |  | 41 | 8.6% | 13.5% | 3.9% | 54.5% |  |  |  |  |  |  |
| Venezuela |  |  |  |  |  |  | 1 | 11.4% | 11.4% | 11.4% | 11.4% |  |  |  |  |  |  |
| **Asia** | **43** | **21.7%** | **20.8%** | **1.1%** | **50.2%** |  | **45** | **15.4%** | **16.8%** | **1.0%** | **47.9%** |  | **6** | **17.60%** | **16.7%** | **4.0%** | **31.4%** |
| China | 7 | 15.5% | 22.2% | 6.2% | 46.8% |  | 4 | 16.6% | 14.6% | 6.7% | 18.5% |  | 2 | 23.90% | 23.9% | 16.4% | 31.4% |
| Gulf cluster* |  |  |  |  |  |  | 1 | 6.4% | 6.4% | 6.4% | 6.4% |  |  |  |  |  |  |
| India | 1 | 11.0% | 11.0% | 11.0% | 11.0% |  | 1 | 10.1% | 10.1% | 10.1% | 10.1% |  |  |  |  |  |  |
| Iran | 1 | 29.1% | 29.1% | 29.1% | 29.1% |  | 4 | 21.4% | 21.6% | 15.4% | 28.3% |  |  |  |  |  |  |
| Israel | 1 | 14.0% | 14.0% | 14.0% | 14.0% |  |  |  |  |  |  |  |  |  |  |  |  |
| Japan |  |  |  |  |  |  | 2 | 41.0% | 41.0% | 37.7% | 44.2% |  |  |  |  |  |  |
| Lebanon | 1 | 8.0% | 8.0% | 8.0% | 8.0% |  |  |  |  |  |  |  |  |  |  |  |  |
| Malaysia | 1 | 7.1% | 7.1% | 7.1% | 7.1% |  |  |  |  |  |  |  |  |  |  |  |  |
| Mongolia | 1 | 23.6% | 23.6% | 23.6% | 23.6% |  | 4 | 21.8% | 24.2% | 14.6% | 36.3% |  |  |  |  |  |  |
| Philippines | 3 | 20.0% | 15.4% | 2.5% | 23.8% |  |  |  |  |  |  |  |  |  |  |  |  |
| Saudi Arabia | 1 | 9.0% | 9.0% | 9.0% | 9.0% |  |  |  |  |  |  |  |  |  |  |  |  |
| Singapore | 6 | 12.0% | 15.9% | 4.9% | 32.5% |  | 1 | 4.5% | 4.5% | 4.5% | 4.5% |  | 1 | 4.00% | 4.0% | 4.0% | 4.0% |
| South Korea | 9 | 25.6% | 23.0% | 1.1% | 29.4% |  | 17 | 13.4% | 12.7% | 1.0% | 29.0% |  | 2 | 20.30% | 20.3% | 18.8% | 21.8% |
| Syria |  |  |  |  |  |  | 1 | 47.9% | 47.9% | 47.9% | 47.9% |  |  |  |  |  |  |
| Taïwan | 2 | 10.5% | 10.5% | 9.6% | 11.3% |  |  |  |  |  |  |  |  |  |  |  |  |
| Thailand | 3 | 32.1% | 27.7% | 13.2% | 37.7% |  | 1 | 5.2% | 5.2% | 5.2% | 5.2% |  | 1 | 7.93% | 7.9% | 7.9% | 7.9% |
| Turkey | 2 | 33.2% | 33.2% | 31.3% | 35.1% |  | 8 | 15.5% | 16.9% | 6.4% | 29.6% |  |  |  |  |  |  |
| United Arab Emirates | 3 | 32.0% | 25.6% | 9.0% | 35.9% |  | 1 | 7.0% | 7.0% | 7.0% | 7.0% |  |  |  |  |  |  |
| Vietnam | 2 | 31.3% | 31.3% | 12.3% | 50.2% |  | 1 | 22.4% | 22.4% | 22.4% | 22.4% |  |  |  |  |  |  |
| **Europe** | **35** | **34.6%** | **32.6%** | **4.1%** | **56.6%** |  | **184** | **18.8%** | **20.1%** | **1.0%** | **43.9%** |  | **6** | **10.60%** | **11.4%** | **5.5%** | **23.5%** |
| Austria |  |  |  |  |  |  | 1 | 16.4% | 16.4% | 16.4% | 16.4% |  |  |  |  |  |  |
| Belgium | 2 | 46.0% | 46.0% | 39.3% | 52.7% |  | 8 | 24.7% | 23.5% | 13.9% | 30.9% |  | 1 | 9.60% | 9.6% | 9.6% | 9.6% |
| Denmark | 3 | 54.1% | 47.4% | 31.6% | 56.6% |  | 22 | 17.9% | 17.4% | 6.5% | 26.1% |  | 1 | 23.50% | 23.5% | 23.5% | 23.5% |
| Estonia | 1 | 39.1% | 39.1% | 39.1% | 39.1% |  | 4 | 18.0% | 16.5% | 9.1% | 21.1% |  |  |  |  |  |  |
| Finland | 1 | 35.7% | 35.7% | 35.7% | 35.7% |  | 14 | 30.4% | 28.5% | 8.1% | 42.3% |  |  |  |  |  |  |
| France | 4 | 27.4% | 25.4% | 4.1% | 42.7% |  | 11 | 21.0% | 23.9% | 8.9% | 43.9% |  |  |  |  |  |  |
| Germany | 1 | 39.6% | 39.6% | 39.6% | 39.6% |  | 13 | 18.1% | 16.9% | 8.3% | 25.0% |  |  |  |  |  |  |
| Greece |  |  |  |  |  |  | 2 | 21.6% | 21.6% | 18.4% | 24.7% |  |  |  |  |  |  |
| Iceland |  |  |  |  |  |  | 3 | 17.8% | 17.5% | 11.2% | 23.6% |  |  |  |  |  |  |
| Ireland |  |  |  |  |  |  | 3 | 20.0% | 19.3% | 14.3% | 23.6% |  |  |  |  |  |  |
| Italy | 5 | 12.9% | 19.8% | 5.6% | 36.1% |  | 21 | 18.3% | 19.2% | 11.1% | 37.7% |  | 1 | 11.60% | 11.6% | 11.6% | 11.6% |
| Macedonia |  |  |  |  |  |  | 1 | 41.3% | 41.3% | 41.3% | 41.3% |  |  |  |  |  |  |
| Netherlands |  |  |  |  |  |  | 6 | 22.2% | 22.6% | 14.4% | 29.5% |  |  |  |  |  |  |
| Norway |  |  |  |  |  |  | 6 | 14.2% | 16.1% | 10.0% | 28.4% |  |  |  |  |  |  |
| Poland | 5 | 32.6% | 32.3% | 27.5% | 36.0% |  | 6 | 20.9% | 20.2% | 4.8% | 36.1% |  |  |  |  |  |  |
| Portugal | 2 | 28.0% | 28.0% | 26.1% | 29.8% |  | 4 | 20.0% | 20.6% | 9.4% | 32.9% |  |  |  |  |  |  |
| Russia |  |  |  |  |  |  | 6 | 4.9% | 7.4% | 1.0% | 26.1% |  |  |  |  |  |  |
| Scotland |  |  |  |  |  |  | 3 | 18.2% | 14.1% | 5.7% | 18.4% |  |  |  |  |  |  |
| Serbia |  |  |  |  |  |  | 1 | 19.4% | 19.4% | 19.4% | 19.4% |  |  |  |  |  |  |
| Spain | 1 | 34.6% | 34.6% | 34.6% | 34.6% |  | 6 | 14.4% | 13.6% | 8.3% | 18.1% |  |  |  |  |  |  |
| Sweden | 7 |  | 35.0% | 19.3% | 51.0% |  | 22 | 24.5% | 23.3% | 7.7% | 30.9% |  | 2 | 9.20% | 9.2% | 6.5% | 12.0% |
| Switzerland |  |  |  |  |  |  | 12 | 16.6% | 17.6% | 11.2% | 25.7% |  | 1 | 5.50% | 5.5% | 5.5% | 5.5% |
| United Kingdom | 3 | 37.0% | 31.6% | 13.7% | 44.0% |  | 9 | 21.8% | 23.6% | 13.2% | 33.2% |  |  |  |  |  |  |
| **Oceania** | **1** | **13.2%** | **13.2%** | **13.2%** | **13.2%** |  | **13** | **38.2%** | **36.5%** | **19.2%** | **47.5%** |  |  |  |  |  |  |
| Australia | 1 | 13.2% | 13.2% | 13.2% | 13.2% |  | 10 | 41.0% | 37.4% | 19.2% | 47.5% |  |  |  |  |  |  |
| New Zealand |  |  |  |  |  |  | 3 | 36.4% | 33.5% | 25.8% | 38.2% |  |  |  |  |  |  |

AR: Allergic Rhinitis; Max: highest reported prevalence; Med: Median; Min: lowest reported prevalence; n: number of reported prevalences; NAR: Non-Allergic Rhinitis.

*Gulf cluster: Kuwait, Saudi Arabia, United Arab Emirates

Table S3: Evolution of rhinitis prevalence over time

| **Country** | **Reference** | **Definition** | **Year: prevalence** |  |
| --- | --- | --- | --- | --- |
| **Americas** | | | |  |
| Brazil | Oliveira *et al.* 2020 | *“Have you ever had sneezing and runny nose in the past 12 months?”* | 2011: 63.3%  2018: 50.5% | ↓ |
|  | Oliveira *et al.* 2020 | *"Have you ever had or have hay fever?"* | 2011: 52%  2018: 43.3% | ↓ |
| United States | NHANES | Episode of hay fever in the past 12 months | 2007-2008: 11.8%  2009-2010: 12.0%  2011-2012: 13.6% | ↑ |
|  | Blackwell *et al.* 2002 Pleis *et al.* 2002 Pleis *et al.* 2003 Pleis *et al.* 2003 Lucas *et al.* 2004 Lethbridge-Çejku *et al.* 2004 Lethbridge-Çejku *et al.* 2005 Lethbridge-Çejku *et al.* 2006 Pleis *et al.* 2006 Pleis *et al.* 2007 Pleis *et al.* 2009 Pleis *et al.* 2009 Pleis *et al.* 2010 Schiller *et al.* 2012 Schiller *et al.* 2012 Blackwell *et al.* 2014 NHIS | “*During the past 12 months, have you been told by a doctor or other health professional that you had hay fever?”* | 1997: 9.3%  1998: 9.0%  1999: 8.9%  2000: 9.3%  2001: 10.0%  2002: 8.8%  2003: 8.6%  2004: 8.6%  2005: 8.6%  2006: 8.0%  2007: 7.6%  2008: 8.0%  2009: 7.8%  2010: 7.8%  2011: 7.3%  2012: 7.5%  2013: 7.6%  2014: 7.7%  2015: 7.9%  2016: 6.2%  2017: 7.7%  2018: 7.3% | ↓ |
| **Asia** | | | | |
| Korea | Myong *et al.* 2012 | *"Have you ever been diagnosed with allergic rhinitis by a doctor?"* | 1998: 1.0%  2001: 2.3%  2005: 7.0%  2007-2009: 10.6% | ↑ |
|  | Ha *et al.* 2020 | *"Have you ever been diagnosed with allergic rhinitis by a doctor?"* | 2008-2009: 13.5%  2013-2015: 16.0%  2016-2017: 17.1% | ↑ |
| **Europe** | | | |  |
| Denmark | Leth‐Møller *et al.* 2019 | a) Itchy or stuffy nose or sneezing when near grass, trees, or flowers within the last 12 months OR b) Itchy or stuffy nose or sneezing when near furry animals within the last 12 months OR c) Itchy or stuffy nose or sneezing when cleaning rooms or making beds, or when in bed within the last 12 months AND sIgE sensitisation | 1990-1991: 6.5%  2006-2008: 15.8%  2012-2015: 17.9% | ↑ |
|  | Leth‐Møller *et al.* 2019 | a) Itchy or stuffy nose or sneezing when near grass, trees, or flowers within the last 12 months OR b) Itchy or stuffy nose or sneezing when near furry animals within the last 12 months OR c) Itchy or stuffy nose or sneezing when cleaning rooms or making beds, or when in bed within the last 12 months AND SPT sensitisation | 2006-2008: 16.7%  2010-2011: 17.0%  2016-2017: 22.0% | ↑ |
|  | Linneberg *et al.* 2000 | Itchy or stuffy nose or sneezing during summer months or when near grass, trees, or flowers or when near furry animals + SPT positive | 1990: 12.9%  1998: 22.5% | ↑ |
|  | Linneberg *et al.* 2000 | Self-reported hay fever | 1990: 16.7%  1998: 23.7% | ↑ |
|  | Linneberg *et al.* 2000  Leth‐Møller *et al.* 2019 | *"Has a physician ever told you that you have hay fever?"* | 1990-1991: 8.0%  1998: 15.6%  2006-2008: 17.9%  2010-2011: 18.7%  2012-2015:18.9%  2016-2017: 20.7% | ↑ |
| Finland | Jousilahti *et al.* 2016 | *"Have you ever had hay fever or other allergic nasal symptoms?" "Yes in the past 12 months"* | 1997: 29.4%  2002: 30.3%  2007: 32.2%  2012: 32.6% | ↑ |
|  | Laatikainen *et al.* 2011 | Hay fever ever occurred, self-reported symptoms/disease | 1997: 21.9%  2007: 30.5% | ↑ |
|  | Laatikainen *et al.* 2011 | *"Has a physician ever told you that you have hay fever?"* | 1997: 8.1%  2007: 13.2% | ↑ |
| France | Annesi-Maesano *et al.* 2002 Klossek *et al.* 2009 | Score for Allergic Rhinitis ≥ 7 | 1997: 21.0%  2007: 31.0% | ↑ |
| Germany | Heinrich *et al.* 1998 | "*Do you have any nasal allergies including hay fever?"* | 1990-1992:  - 13.3% Est Germany  -22.9% West Germany  1994-1995:  - 15.9% Est Germany  - 24.6% West Germany | ↑ |
| Italy | Marco *et al.* 2011 | "*Do you have any nasal allergies including hay fever?*" | 1991-1993: 16.8%  1998-2000: 19.4%  2007-2010: 25.8% | ↑ |
| Poland | Sozańska *et al.* 2014 | *"Problem with sneezing or a runny or blocked nose or itchy eyes in April, May, June, or July"* | 2003: 30.0%  2012: 27.5% | ↓ |
|  | Sozańska *et al.* 2014 | *"In last 12 mo, have you had a problem with sneezing or a runny or blocked nose or itchy eyes when you (your child) did not have a cold or flu?"* | 2003: 35.2%  2012: 32.6% | ↓ |
|  | Sozańska *et al.* 2014 | *"Has a physician ever told you that you have hay fever?"* | 2003: 4.8%  2012: 7.7% | ↑ |
| Russia | Laatikainen *et al.* 2011 | Ever hay fever | 1997-1998 : 4.2%  2007: 5.8% | ↑ |
|  | Laatikainen *et al.* 2011 | *"Has a physician ever told you that you have hay fever?"* | 1997-1998: 1.0%  2007: 5.6% | ↑ |
| Scotland | Upton *et al.* 2000 | *“Do you suffer from, or have you ever suffered from, hay fever?”* | 1972-1976: 5.73%  1996: 18.4% | ↑ |
| Sweden | Nihlén *et al.* 2006 | *"Do you have or do you have had hay fever?"* and *"Do you have nasal symptoms either permanently or recurrently?”* Moreover, a report was required that at least one of the following four environmental factors provoked nasal symptoms: tree-, grass pollen, furred animals, or house dust. | 1992: 12.4%  2000: 15% | ↑ |
|  | Nihlén *et al.* 2006 | *"Do you have or do you have had hay fever?”* | 1992: 20.5%  2000: 25.0% | ↑ |
|  | Bjerg *et al.* 2011 | *“Do you have any nasal allergies including hay fever?’’* | 1990: 21.6%  2008: 30.9% | ↑ |
|  | Borna *et al.* 2019 | *“Have you now, or have you ever had allergic rhinitis (hay‐fever)?”* | 2008: 26.9%  2016: 28.6% | ↑ |
| **Oceania** | | | |  |
| Australia | Peat *et al.* 1992 | Hay fever in the previous 12 months | 1981: 21.9%  1990: 46.7% | ↑ |
